# Rhenium in Wild Mushrooms: Environmental Occurrence and Censoring-Aware Quantification

**DOI:** 10.3390/ijms27188405

**Published:** 2026-09-21

**Authors:** Antonio Peña-Fernández, Borja Martínez-Alonso, Rafael Moreno-Gómez-Toledano, Tomás Cámara-Pastor

**Affiliations:** 1 Area of Legal and Forensic Medicine, Department of Surgery, Medical and Social Sciences, Faculty of Medicine and Health Sciences, University of Alcalá, Ctra. Madrid-Barcelona Km, 33.6, 28871 Alcalá de Henares, Spain; 2 Leicester School of Allied Health Sciences, De Montfort University, Leicester LE1 9BH, UK; 3 Department of Biomedical Sciences, Faculty of Pharmacy, University of Alcalá, Crta. Madrid-Barcelona Km, 33.6, 28871 Alcalá de Henares, Spain; 4 Area of Human Anatomy and Embryology, Faculty of Medicine and Health Sciences, University of Alcalá, Crta. Madrid-Barcelona Km, 33.6, 28871 Alcalá de Henares, Spain; 5 Scientific Computation Research Institute (SCRIUR), University of La Rioja, 26006 Logroño, Spain

**Keywords:** rhenium, wild mushrooms, technology-critical metals, environmental occurrence, environmental biomonitoring, ICP–MS, interval censoring, censoring-aware statistics

## Abstract

Rhenium (Re) is a scarce technology-critical transition metal used in high-temperature superalloys, catalysts and other advanced materials, yet its occurrence in terrestrial biota and potential relevance to environmental exposure remains poorly characterised. This study investigated total Re in wild mushroom fruiting bodies collected from selected green spaces in Leicester and Leicestershire, United Kingdom, using inductively coupled plasma mass spectrometry (ICP–MS) and censoring-aware statistical analysis. The analytical dataset comprised 157 records. After exclusion of four unmatched tissue-only records, 153 analytical portions were reconstructed into 121 fruiting-body-level biological units, avoiding pseudoreplication from separately analysed cap and stem tissues. The analysis incorporated procedural digestion blanks, sample-specific dry-weight analytical limits and interval censoring. Under the primary analytical scenario, 73/121 units (60.3%) were non-detects, 21 (17.4%) were detected below the operational lower limit of quantification (LLOQ), seven (5.8%) were partially quantified and 20 (16.5%) were fully quantified. Overall, 48/121 units (39.7%) contained at least one Re signal above the operational detection threshold, whereas 27/121 (22.3%) contained at least one quantitatively valid component. Fully quantified concentrations ranged from 3.91 to 163.70 ng g^−1^ dry weight. The complete dataset comprised 73 left-censored, 28 interval-censored and 20 exact observations. An interval-censored lognormal model estimated a population median of 1.055 ng g^−1^ dry weight (95% bootstrap confidence interval: 0.672–1.571), with all 1000 bootstrap refits converging successfully. Classification was identical for all 59 units directly comparable under the primary and stricter same-batch procedural-background definitions. These findings extend the limited evidence for Re occurrence in wild fungal fruiting bodies and should be interpreted as environmental-occurrence data rather than as evidence of either adverse risk or safety. Because total elemental Re does not resolve chemical form, gastrointestinal bioaccessibility or internal dose, the measured concentrations do not support quantitative estimates of dietary exposure or human-health risk.

## 1. Introduction

Rhenium (Re; atomic number 75) is among the least abundant naturally occurring elements in the continental crust and is generally present at very low concentrations in common environmental matrices [1,2]. In contrast to its geochemical scarcity, Re has considerable technological importance because of its exceptionally high melting point, thermal stability and favourable performance in refractory metallic systems. Principal applications include nickel-based high-temperature superalloys used in aerospace and gas-turbine components and platinum–rhenium catalysts employed in petroleum refining, together with more specialised applications in thermocouples, electrical contacts, X-ray targets and advanced coatings [1]. Extraction, refining, high-performance manufacturing, catalyst use, recycling and end-of-life management consequently place Re within increasingly complex anthropogenic material cycles.

The environmental behaviour of Re is strongly influenced by oxidation state. Under oxic conditions, Re(VII) commonly occurs as the perrhenate oxyanion (ReO_4_^−^), a relatively soluble and environmentally mobile species in aqueous and near-surface systems [2,3,4]. Tagami and Uchida reported geometric mean total Re concentrations of approximately 0.34, 0.23 and 0.28 ng g^−1^ in Japanese paddy, upland and other agricultural soils, respectively, whereas the corresponding water-soluble fractions varied substantially among soil types [3]. Water-soluble Re represented approximately 16% of total Re in paddy soils, 6% in upland soils and 3% in other soils, demonstrating that bulk concentration alone does not define the potentially mobile fraction [3]. The chemical similarity of perrhenate and pertechnetate has also led to the use of stable Re as an analogue for technetium in environmental transport studies [3,4].

Evidence from vascular plants confirms that environmentally available Re can enter terrestrial biological systems. Re and Tc showed comparable uptake and distribution behaviour in radishes exposed through nutrient solution [4]. Re accumulation has also been demonstrated in vegetation associated with Cu–Mo mineralisation and in experimental systems developed for phytomining [5,6]. Tzvetkova et al. reported substantial Re accumulation in alfalfa leaves, with a large fraction recoverable by water extraction, consistent with the presence of a readily soluble Re form [5]. More recently, controlled exposure of *Lactuca sativa* to Re-enriched growth media produced concentration-dependent uptake and physiological effects, including marked growth inhibition at the highest experimental exposures [7]. These experimentally enriched conditions were substantially above those generally encountered in environmental background settings and cannot be extrapolated quantitatively to field biomonitoring data. Nevertheless, they demonstrate that Re can become biologically accessible under the appropriate chemical conditions.

Wild mushrooms provide a complementary and comparatively underused matrix for investigating terrestrial element occurrence. Fruiting-body elemental composition reflects interactions among substrate geochemistry, element bioavailability, fungal identity and physiology, trophic or ecological strategy, mycelial distribution and microscale environmental heterogeneity. Studies of wild fungi further indicate that bulk soil or substrate concentrations may be only weak-to-moderate predictors of concentrations in fruiting bodies, with species-specific accumulation behaviour and soil physicochemical properties substantially modifying elemental profiles [8,9,10]. This distinction is important because the mycelial system may exploit spatially heterogeneous substrate domains that are not adequately represented by a single bulk or composite soil measurement.

Within the broader environmental literature, particular attention is given to potentially toxic elements (PTEs) and other trace elements of toxicological significance because environmental occurrence does not necessarily translate directly into biological exposure or adverse effect. Their environmental behaviour is controlled by a combination of geogenic background and anthropogenic inputs, together with element-specific speciation, mobility, sorption, bioavailability and biological uptake. Some trace elements may be essential at physiological concentrations yet become toxicologically relevant at elevated exposures, whereas others have no established biological requirement. Consequently, interpretation requires consideration of both concentration and chemical form rather than elemental occurrence alone. Wild fungi are useful biomonitoring matrices in this context because they can accumulate trace elements selectively, and fruiting-body concentrations may differ substantially from those predicted from bulk-soil concentrations alone as a result of fungal identity, substrate characteristics and element bioavailability [8,9,10]. Rhenium can be placed within this wider framework of environmentally relevant trace elements, but its behaviour should not be inferred directly from that of more extensively studied PTEs. Under oxic conditions, Re commonly occurs as the relatively soluble perrhenate oxyanion, giving it environmental mobility and bioavailability characteristics that differ from those of many cationic metallic elements [2,3,4]. Its toxicological significance in environmental and dietary settings also remains much less defined than that of established PTEs, making careful separation of environmental occurrence, chemical speciation, exposure and biological effect particularly important.

Direct evidence for Re occurrence in mushrooms remains exceptionally limited. Mleczek et al. quantified Re within a 26-element survey of 20 wild mushroom species collected near a heavily trafficked road in Poland, with the highest reported aboveground concentration occurring in *Tricholoma equestre* [11]. Re has also been reported in commercially cultivated *Lentinula edodes* [12], in fungi from the Re-rich Zhireken Cu–Mo mineralised environment [13], and in earlier exploratory work involving *Macrolepiota procera* and *Armillaria/Armillariella mellea* [14]. Collectively, these studies establish fungal incorporation of Re but provide little information on its occurrence in wild mushrooms collected from non-mineralised public green spaces under routine environmental-monitoring conditions.

The toxicological significance of environmentally encountered Re cannot be inferred from total elemental concentration alone. Information relevant to chronic environmental or dietary exposure remains sparse, and the available evidence indicates substantial dependence on chemical form [1,15,16]. Early experimental studies reported comparatively low acute toxicity for soluble perrhenate salts under the conditions examined, whereas other inorganic, organometallic and coordination compounds of Re exhibit distinct toxicological profiles [1,15,16]. Perrhenate is nevertheless biologically transportable and is recognised by the sodium–iodide symporter (NIS), enabling uptake by NIS-expressing tissues [17]. Chemical speciation is therefore central to any biologically meaningful interpretation of environmental Re occurrence.

This distinction is especially important when fungal Re is considered in relation to potential dietary exposure. Total trace-element concentration does not identify the chemical species released from the food matrix during digestion, the gastrointestinally bioaccessible fraction or the proportion subsequently available for systemic absorption [18,19,20]. Studies of edible mushrooms have demonstrated that both speciation and gastrointestinal bioaccessibility can materially alter the interpretation of exposure based on total concentrations [18,19,20]. Contemporary risk-assessment frameworks likewise distinguish external exposure from absorption, toxicokinetics, internal dose and ultimately biologically effective dose and molecular response [21]. Environmental occurrence is therefore an essential starting point for exposure assessment, but it is not itself a measure of absorbed dose or toxicity.

A separate mechanistic literature concerns deliberately synthesised Re-containing compounds, including Re(I) tricarbonyl complexes, Re clusters and related coordination compounds developed primarily for biomedical applications [22,23,24,25,26,27,28,29,30,31,32,33]. These studies demonstrate that defined Re species can engage diverse cellular pathways and that biological activity depends strongly on chemical form, molecular structure, cellular uptake and subcellular localisation [16,22,23,24,25,26,27,28,29,30,31,32,33]. However, these pharmacological and compound-specific findings provide only external mechanistic context and hypothesis-generating evidence; they cannot be extrapolated quantitatively to ultratrace total elemental Re measured in environmental mushroom samples.

Against this background, the present study aimed to: (i) reconstruct a traceable fruiting-body-level Re dataset from the original analytical records; (ii) characterise Re detection and quantitative coverage using study-specific, sample-specific operational analytical limits; (iii) estimate the underlying Re concentration distribution using censoring-aware methods without arbitrary fixed-value substitution; (iv) evaluate the stability of analytical classification under alternative procedural-background definitions; (v) characterise location-specific and taxonomic occurrence while explicitly accounting for the inferential constraints of the unbalanced sampling design; and (vi) place fungal Re occurrence within the broader context of environmental mobility, exposure-relevant uncertainties, chemical speciation and external toxicological literature relevant to hypothesis generation.

## 2. Results

### 2.1. Dataset Reconstruction and Analytical Performance

**Figure 1:**
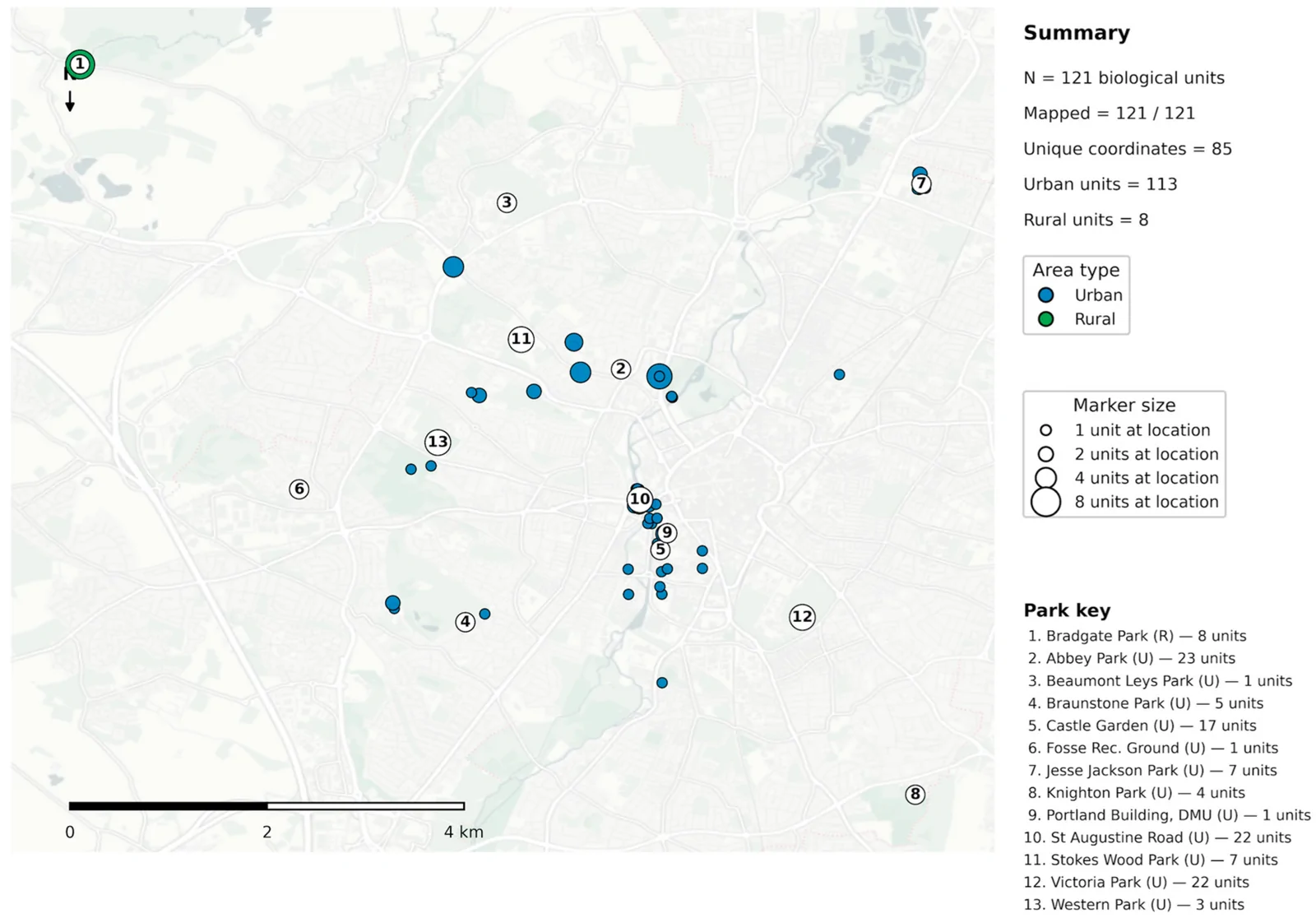
Mushroom sampling locations. Spatial distribution of the 121 reconstructed mushroom fruiting-body units included in the study. Marker size is proportional to the number of fruiting-body units represented at each mapped location, and colours distinguish urban and rural sampling areas. The figure is provided to show the geographical distribution of mushroom sampling locations and was not used for traffic-proximity or source-apportionment analysis.

The original dataset comprised 157 mushroom analytical records, including whole-fruiting-body measurements and separately analysed cap and stem portions. Four unmatched tissue-only records could not be reconstructed into complete fruiting-body units and were therefore excluded from the principal biological dataset. The remaining 153 analytical portions comprised 89 whole-fruiting-body records and 64 tissue-specific records corresponding to 32 matched cap–stem pairs. Reconstruction yielded 121 fruiting-body-level biological units, which constituted the principal unit of analysis. The geographical distribution of the 121 reconstructed fruiting-body units across the sampling locations is shown in Figure 1. The map is provided for spatial context and was not used for traffic-proximity or source-apportionment analysis.

Rhenium was monitored as ^187^Re by ICP–MS. External calibration covered 0.005–0.137 µg L^−1^ and showed high linearity (*R*^2^ = 0.999476). The lowest calibration standard (0.005 µg L^−1^) had a relative standard deviation (RSD) of 7.15% and a back-calculation error of −14.05%. In the absence of a formal matrix-specific metrological validation of the quantification limit, this concentration was retained and reported throughout as the study-specific operational LLOQ. Detailed calibration performance data are provided in Table S1.

The primary procedural-background population comprised eight procedure-defined post-replacement acid-digestion blanks and yielded a Scenario A operational detection threshold of 0.001046 µg L^−1^ on the introduced-solution scale.

An independently prepared aqueous Re reference solution produced an RSD of 3.03% in triplicate analysis. Because this control was analysed outside the calibration interval used for the environmental samples and did not undergo the mushroom digestion procedure, it was interpreted as an assessment of instrumental repeatability rather than as evidence of matrix recovery or analytical trueness.

Variation in analytical-portion mass resulted in sample-specific dry-weight detection and quantification limits after conversion from the common solution-scale limits.

The principal analytical-performance and quality-assurance parameters for ^187^Re determination are summarised in Table 1.

### 2.2. Re Occurrence and Censoring-Aware Concentration Distribution

Under Scenario A, 73/121 fruiting-body units were classified as non-detects (60.3%; exact 95% CI: 51.0–69.1%). Twenty-one units (17.4%; 95% CI: 11.1–25.3%) contained Re signals above the operational detection threshold but below the operational LLOQ, seven units (5.8%; 95% CI: 2.4–11.6%) were partially quantified, and 20 units (16.5%; 95% CI: 10.4–24.4%) were fully quantified.

**Figure 2:**
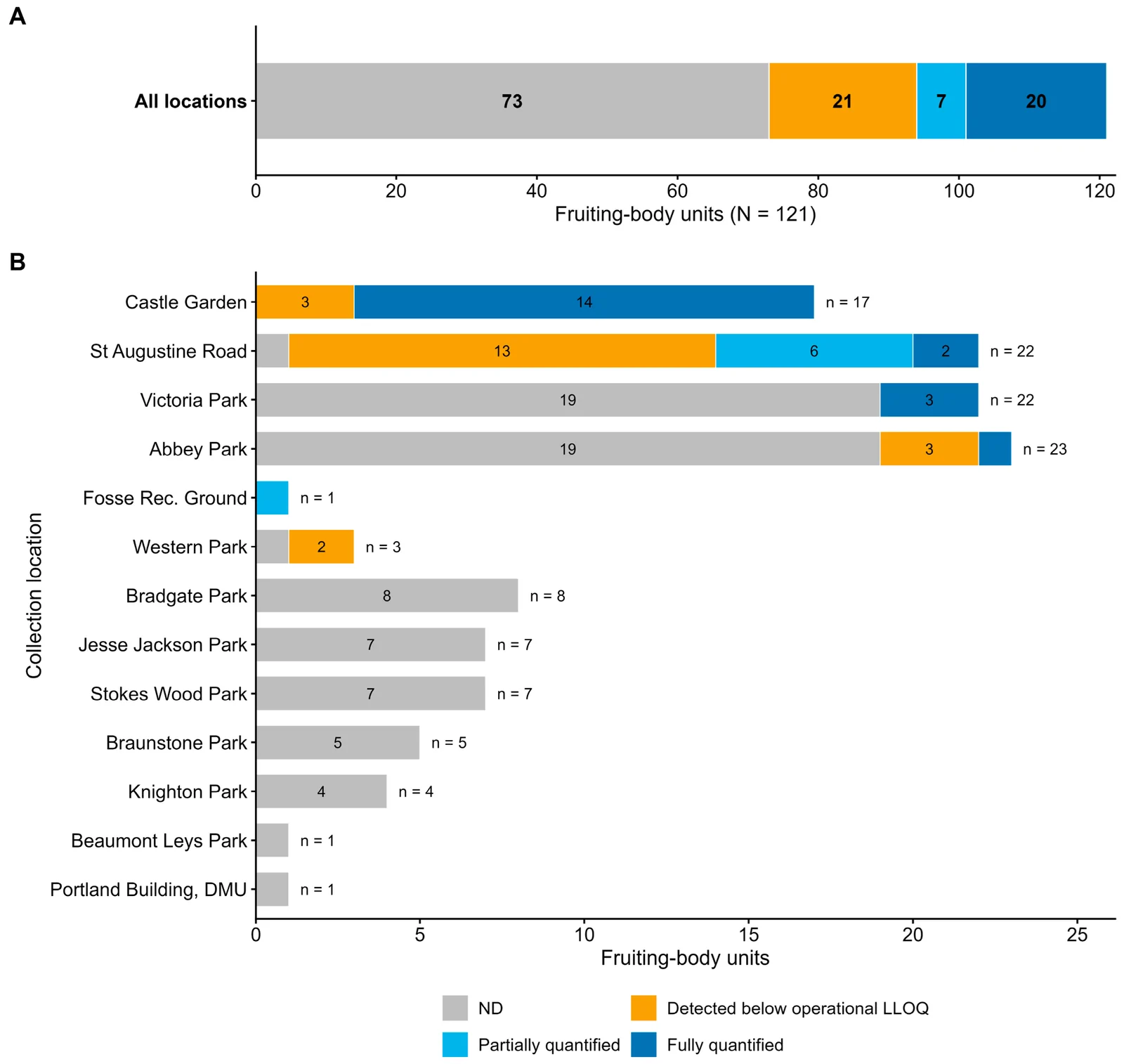
Re occurrence by sampling location. (**A**) Global analytical-classification distribution among the 121 reconstructed fruiting-body units. (**B**) Location-specific count distributions using the same four analytical classes. Counts rather than percentages are shown, and *n* denotes the total number of fruiting-body units at each location. Location-specific patterns are descriptive because sampling intensity, taxonomic composition and analytical-batch representation were unbalanced among sites.

Overall, 48/121 fruiting-body units (39.7%) contained at least one Re signal above the operational detection threshold, whereas 27/121 (22.3%) contained at least one quantitatively valid component. The complete occurrence and censoring profile is summarised in Table 2. The overall distribution of Scenario A analytical classifications and their descriptive distribution across collection locations are shown in Figure 2.

The individual fully and partially quantified fruiting-body units, together with their exact concentrations or reconstructed analytical intervals, are reported in Table S2.

**Figure 3:**
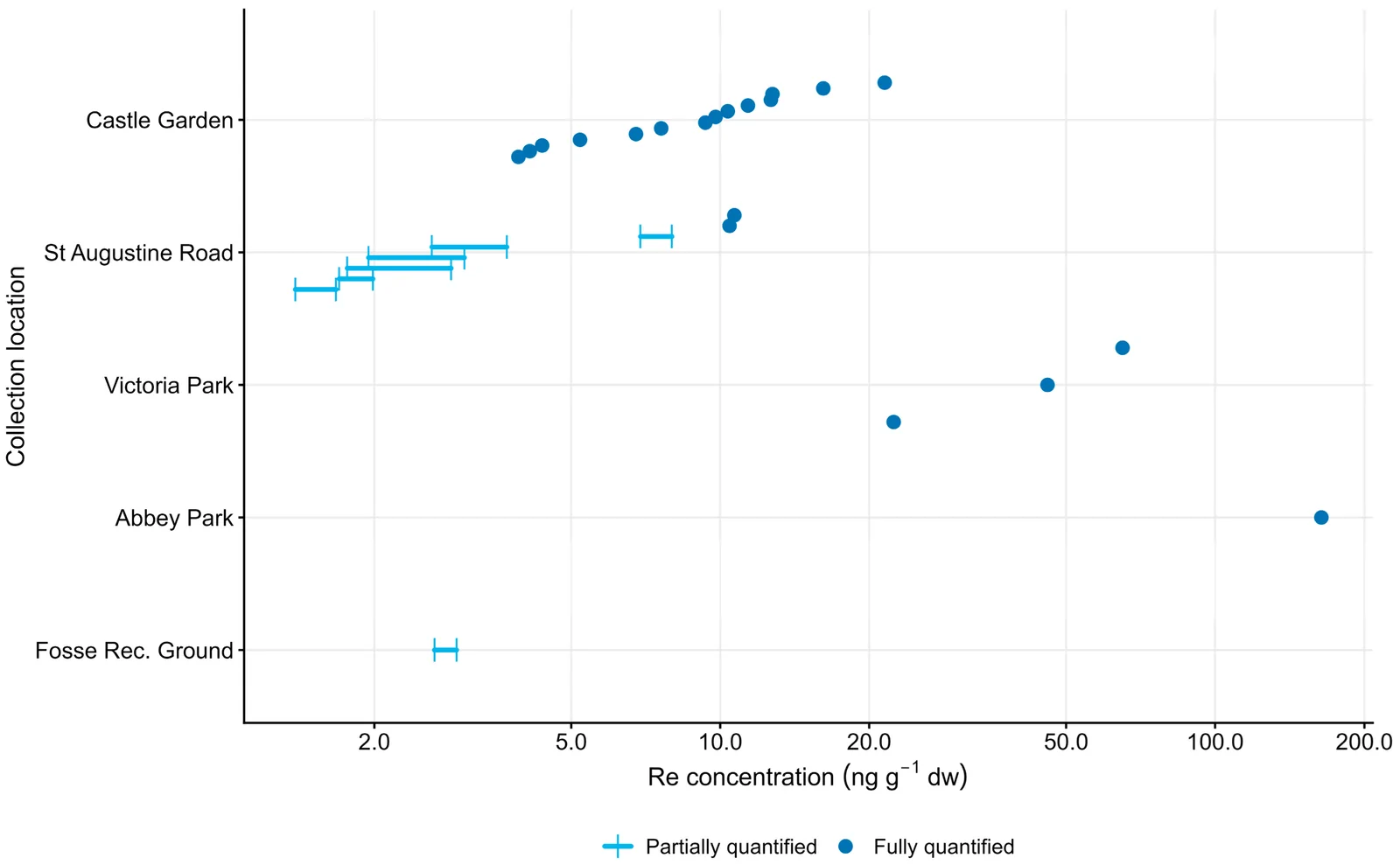
Re concentrations among quantitatively informative fruiting bodies. The plot includes the 27 fruiting-body units with at least one quantitatively valid component: 20 fully quantified units shown as exact concentrations and seven partially quantified units shown as lower–upper analytical intervals. Units are grouped by collection location and concentrations are displayed on a logarithmic scale. No midpoint or other fixed-value substitution was applied to interval-censored observations.

Among the 20 fully quantified units, Re concentrations ranged from 3.910 to 163.705 ng g^−1^ dw, with a median of 10.562 ng g^−1^ dw (interquartile range [IQR]: 7.388–17.491 ng g^−1^ dw). This statistic describes only the exact subset and was not interpreted as an estimate of the complete population distribution because it necessarily excludes all censored observations. The 27 fruiting-body units containing at least one quantitatively valid component are visualised in Figure 3, with exact concentrations shown for fully quantified units and lower–upper analytical intervals retained for partially quantified units.

Archived sample-specific instrumental precision was available for all 22 analytical component records contributing to the 20 fully quantified fruiting-body units. The ^187^Re instrumental RSD ranged from 44.22% to 183.07% (median 110.32%; mean 108.28%), indicating substantial replicate variability at these ultratrace analyte responses. By contrast, the corresponding ^115^In internal-standard RSDs ranged from 0.55% to 4.17% (median 1.81%; mean 2.03%), indicating stable internal-standard response across the same analytical records. Individual concentrations, derived instrumental SDs and archived ^187^Re RSDs are reported in Table 2, with expanded analytical-record and internal-standard precision information provided in Table S2. The designation “fully quantified” therefore denotes an exact concentration within the study-specific calibration and censoring framework; it does not imply uniformly high instrumental precision. The substantial ^187^Re replicate variability observed for several measurements, despite comparatively stable ^115^In responses, is consistent with the analytical challenges of quantifying Re at these ultratrace signal levels.

The complete Scenario A dataset comprised 73 left-censored, 28 interval-censored and 20 exact observations. Thus, 101/121 observations (83.5%) were non-exact, indicating that complete-case summaries or analyses based on fixed-value substitution would not adequately represent the full concentration distribution.

The intercept-only lognormal interval-censored model converged successfully and estimated a model-based population median of 1.0548 ng g^−1^ dw. Non-parametric bootstrap resampling at the fruiting-body level produced 1000/1000 successful model fits and a 95% confidence interval of 0.6724–1.5711 ng g^−1^ dw. No fixed numerical substitution was applied to censored observations in deriving the primary distributional estimate.

Distributional sensitivity analysis showed that the primary median estimate was robust across the two best-supported parametric forms. The log-logistic model provided a marginally lower AIC and BIC (AIC = 362.04; BIC = 367.63) than the lognormal model (AIC = 362.58; BIC = 368.17; ΔAIC = 0.54), while yielding an almost identical estimated median of 1.066 ng g^−1^ dw compared with 1.055 ng g^−1^ dw for the lognormal model. Weibull and gamma models received substantially less support (AIC = 370.46 and 384.37, respectively) and yielded lower estimated medians of 0.873 and 0.799 ng g^−1^ dw, respectively. Thus, although estimates varied across less-supported distributional forms, the central estimate was essentially unchanged between the two models receiving strongest support from the data.

### 2.3. Location-Specific and Taxonomic Patterns of Re Occurrence

The distribution of Scenario A analytical classifications varied substantially among collection locations. These patterns were interpreted descriptively because sampling intensity, taxonomic composition and analytical-batch representation were unbalanced among sites and did not permit independent estimation of location effects. The corresponding location-specific distributions are shown in Figure 2B.

Castle Garden contained 17 reconstructed units, of which 14 were fully quantified and three were detected below the operational LLOQ. Thus, all 17 units showed analytical evidence of Re occurrence, and 14/17 (82.4%) contained at least one quantitatively valid component. These 17 fruiting-body units corresponded to the complete subset for which repeated PCR attempts did not yield an ITS amplicon suitable for sequencing and were therefore retained as unknown/unidentified taxa. All Castle Garden units were also analysed within analytical batches B01 and B02. Consequently, collection location, taxonomic status and analytical batch were structurally confounded at this site, and the pronounced Castle Garden Re profile cannot be attributed independently to a location effect, fungal taxon or batch-related analytical context. The Castle Garden pattern is therefore interpreted descriptively and as hypothesis-generating rather than as evidence of an independent site-specific effect.

At St Augustine Road, 21/22 units (95.5%) showed analytical evidence of Re occurrence: 13 were detected below the operational LLOQ, six were partially quantified and two were fully quantified, while one unit was classified as an ND. Eight of the 22 units (36.4%) contained at least one quantitatively valid component.

Victoria Park contributed three fully quantified units among 22 sampled fruiting bodies. Abbey Park contained one fully quantified unit and three additional detections below the operational LLOQ among 23 units. The single unit collected from Fosse Recreation Ground was partially quantified, whereas two of the three Western Park units were detected below the operational LLOQ.

No Scenario A Re signal exceeded the operational detection threshold in fruiting-body units collected from Beaumont Leys Park, Bradgate Park, Braunstone Park, Jesse Jackson Park, Knighton Park, Portland Building DMU or Stokes Wood Park. Detailed location-level occurrence profiles are provided in Table S3.

Among named taxa, *Agaricus bitorquis* accounted for 22 units. Thirteen were detected below the operational LLOQ, six were partially quantified, two were fully quantified and one was an ND. The two *Agaricus arvensis* units comprised one below-LLOQ detection and one partially quantified observation. Three of 22 *Mycena citrinomarginata* units were fully quantified, while the remaining 19 were ND. Among 28 *Panaeolina foenisecii* units, one was fully quantified, three were detected below the operational LLOQ and 24 were ND. One of two *Xerocomus porosporus* units contained a below-LLOQ Re signal.

**Figure 4:**
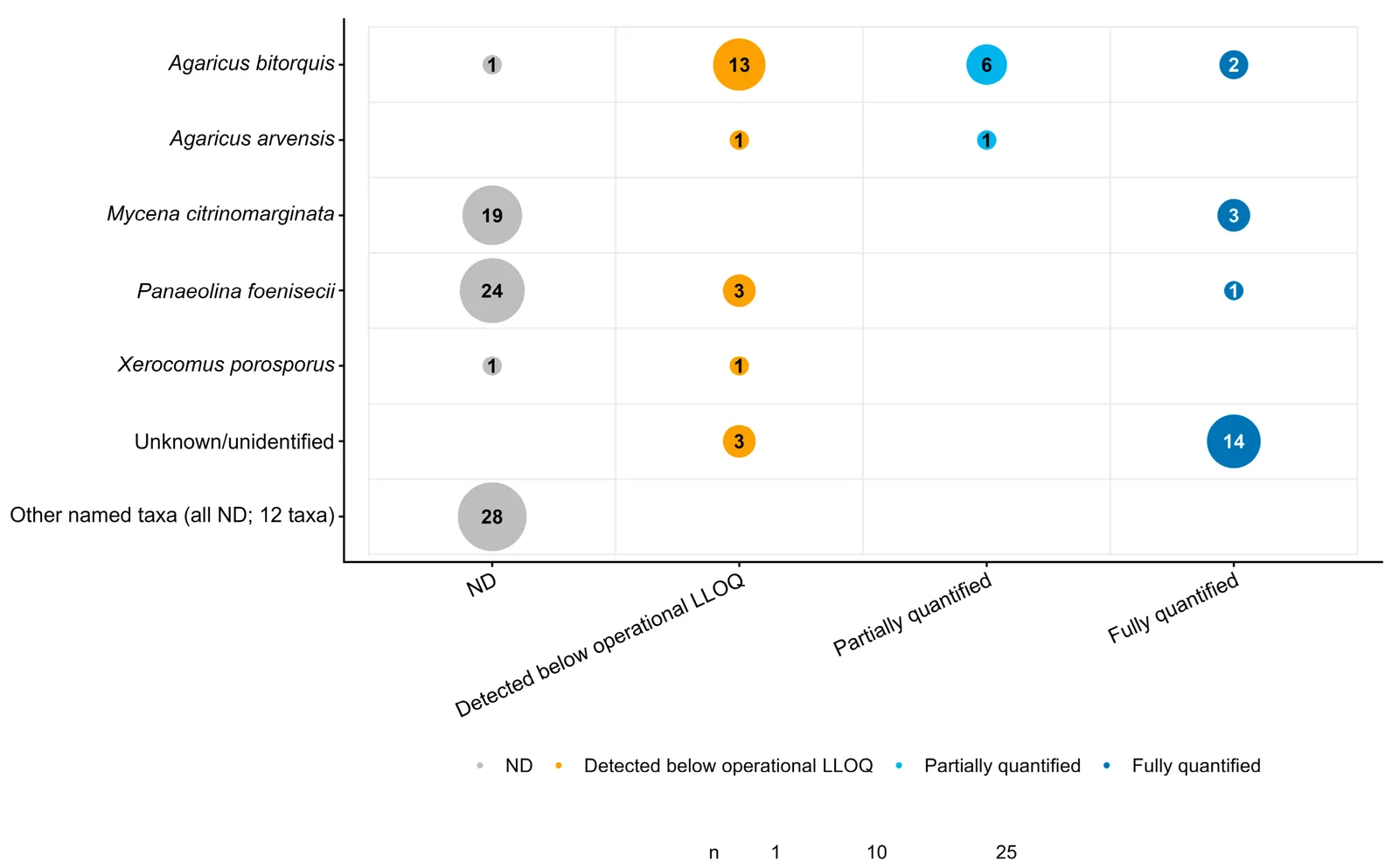
Descriptive taxonomic Re profile across the complete *N* = 121 dataset. Bubble size and the number displayed within each bubble indicate the number of fruiting-body units in the corresponding analytical class. The row ‘Other named taxa (all ND; 12 taxa)’ aggregates the remaining 28 named fruiting-body units, all of which were non-detects. This visualization is descriptive only; no inferential comparison between taxa is intended because collection location, taxon and analytical batch are partially confounded.

The complete descriptive taxonomic profile, including taxa for which all observations were classified as ND, is summarised visually in Figure 4.

These taxonomic profiles are descriptive because several taxa were concentrated within particular collection locations and/or analytical batches. The present sampling design therefore does not permit independent estimation of intrinsic species-specific Re accumulation. Detailed taxon-level occurrence profiles are provided in Table S4.

### 2.4. Cap–Stem Reconstruction

Thirty-two fruiting bodies were represented by matched cap and stem analytical portions. Only two pairs yielded quantitatively exact results for both components. Seven additional pairs contained one quantitatively exact component but did not support an exact reconstructed fruiting-body concentration, whereas the remaining 23 pairs contained no exact component.

The available data therefore did not support an inferential comparison of Re concentrations between cap and stem tissues. Instead, the paired measurements were used to reconstruct fruiting-body-level exact concentrations or censoring intervals while retaining the analytical information contributed by both components.

Reconstruction was weighted using the recorded dry masses of the analytical portions. These masses represent the quantities introduced into digestion and should not be interpreted as experimentally determined whole-fruiting-body cap:stem biomass ratios.

### 2.5. Sensitivity to Analytical-Background Definition

Four analytical-background interpretations were evaluated to determine the sensitivity of fruiting-body classification to alternative treatments of procedural and instrumental background.

Scenario A, the primary analysis, used the procedure-defined population of eight post-replacement acid-digestion blanks and classified 73 units as ND, 21 as detected below the operational LLOQ, seven as partially quantified and 20 as fully quantified.

Scenario B applied the stricter requirement for an eligible post-replacement procedural blank from the same analytical batch and was evaluable for 59/121 units. Fifty-six of these units were ND and three were fully quantified. Classification was identical to Scenario A for all 59 directly comparable units, corresponding to 100% concordance. The remaining 62 units were not evaluable under Scenario B because no eligible same-batch post-replacement procedural blank was available.

Scenario C incorporated all 18 archived acid-digestion blanks, including the earlier procedural phase characterised by elevated blank signals, and was retained as a deliberately conservative sensitivity boundary. Under this definition, 120/121 units were classified as ND and one was partially quantified.

Scenario D used wash-derived instrumental background as a diagnostic alternative. It classified 73 units as ND, 22 as detected below the operational LLOQ, six as partially quantified and 20 as fully quantified, closely reproducing the overall Scenario A classification pattern.

Complete classification counts across Scenarios A–D are reported in Table S6.

Taken together, the sensitivity analyses show that classification of ultratrace Re signals was influenced by analytical-background definition, particularly when the earlier blank phase was incorporated. Nevertheless, classification remained unchanged for all 59 units directly comparable between Scenarios A and B, demonstrating concordance between the primary analysis and the stricter same-batch procedural-background criterion.

### 2.6. Contextual Composite-Topsoil Dataset

The wider environmental-monitoring programme comprised 850 surface-topsoil increments, which were pooled within sampling locations and homogenised to produce 26 site-level composite topsoils. Two separately identified physical subsamples were retained from each composite, yielding 52 analytical records for laboratory QA/QC and sensitivity assessment while preserving *N* = 26 as the environmental sample size.

For Re, final classification used a study-specific operational detection threshold of 0.0718 µg L^−1^ on the 11-fold-diluted analytical-solution scale, corresponding to approximately 79.0 ng g^−1^ dw under the documented soil-preparation conditions. All 52 physical subsamples were below this threshold. Consequently, no quantitatively resolved Re concentration could be assigned to any of the 26 site-level composites.

Nine mushroom-collection sites had an eligible corresponding site-level topsoil composite based on exact site-name matching or previously validated site aliases. All nine matched soil composites were below the operational Re detection threshold. In contrast, mushroom-associated Re signals were observed at four of these nine matched sites—Castle Garden, Abbey Park, Victoria Park and Western Park. Because the soil dataset was entirely left-censored at the operational threshold, quantitative soil–mushroom correlations, concentration ratios, bioconcentration factors and transfer factors were not calculated.

The composite topsoils provide park-scale geochemical context rather than measurements of the specific substrates accessed by individual fungal mycelia. They were not collected directly beneath individual fruiting bodies and were therefore not treated as spatially paired soil–fungus samples. The soil dataset was retained exclusively as contextual environmental information for the broader monitoring programme. Its structure and analytical status are summarised in Table S7.

## 3. Discussion

### 3.1. Environmental Occurrence of Re in Wild Mushrooms

The present study provides evidence of Re occurrence in an opportunistically collected sample of wild mushroom fruiting bodies from selected green spaces in Leicester and Leicestershire that were not chosen on the basis of known Re mineralisation. Under the primary analytical interpretation, Re signals above the operational detection threshold were observed in 48/121 reconstructed fruiting-body units, comprising 20 fully quantified, seven partially quantified and 21 detected-below-operational-LLOQ observations. Preserving these distinct analytical states is particularly important for an ultratrace dataset: reducing the data to a binary detected/non-detected classification would discard information on quantitative status, whereas assigning exact concentrations to sub-LLOQ observations would overstate analytical certainty.

Published information on Re concentrations in fungal fruiting bodies remains remarkably sparse. Mleczek et al. [11] quantified Re as part of a 26-element survey of 20 wild mushroom species collected near a heavily trafficked road in Poland, reporting a maximum aboveground concentration of 0.21 ± 0.05 mg kg^−1^ dw in *Tricholoma equestre*. Their study also revealed substantial interspecific variation, illustrating the importance of fungal identity when comparing elemental occurrence among studies.

Commercially cultivated *Lentinula edodes* has likewise been reported to contain Re, with a general mean concentration of approximately 0.24 mg kg^−1^ dw [12]. This finding confirms Re occurrence in a mushroom entering the food market, although direct comparison with opportunistically collected wild fungi is constrained by differences in fungal taxon, cultivation substrate and production environment.

Substantially higher and more heterogeneous Re concentrations have been reported from geochemically enriched environments. In the Re-rich Zhireken Cu–Mo mineralised area, Safonov et al. [13] reported concentrations of 0.23 mg kg^−1^ dw in a *Leccinum versipelle* cap, 0.054 mg kg^−1^ dw in *Xerocomus subtomentosus* and 1.91 mg kg^−1^ dw in *Suillus elegans*. These observations demonstrate fungal incorporation of Re under mineralised conditions but should not be regarded as representative of environmental background.

The fully quantified range observed in the present study, 0.00391–0.16370 mg kg^−1^ dw, therefore overlaps part of the concentration domain previously reported for mushrooms while remaining below the highest values documented in mineralised settings. Formal cross-study comparison is not warranted, however, because the available studies differ substantially in fungal composition, substrate characteristics, environmental setting, analytical methodology, detection and quantification limits, and treatment of censored observations. Published mushroom Re concentrations relevant to contextual interpretation of the present dataset are summarised in Table 3.

The earlier study by Busuioc et al. [14] provides additional historical evidence for Re occurrence in macromycetes but is less suitable as a quantitative comparator because of substantial differences in analytical sensitivity and reporting.

Marked location-specific heterogeneity was also evident within the present dataset. Castle Garden contributed 14 of the 20 fully quantified fruiting-body units, and all 17 samples collected at this location contained evidence of Re above the operational detection threshold. This marked heterogeneity is also evident in the location-specific occurrence profiles shown in Figure 2B. This pattern is noteworthy but cannot be interpreted as an independent location effect because all 17 Castle Garden specimens belonged to the subset for which ITS amplification was unsuccessful, and location, taxonomic identity and analytical batch were not independently distributed. Similarly, the uneven spatial representation of named taxa precludes attribution of the observed concentration profiles to intrinsic species-specific accumulation. The location-specific and taxonomic patterns should therefore be regarded as hypothesis-generating observations that identify priorities for future balanced sampling rather than as evidence of causal site or species effects. The descriptive taxonomic distribution shown in Figure 4 further illustrates this heterogeneity, but should not be interpreted as evidence of intrinsic species-specific Re accumulation.

### 3.2. Environmental Mobility and Biological Availability

The occurrence of Re in fungal fruiting bodies is compatible with the known environmental behaviour of oxidised Re. Under oxic conditions, Re(VII) commonly occurs as perrhenate (ReO_4_^−^), a soluble oxyanion whose retention is influenced by sorbent chemistry, electrostatic interactions, ionic strength, pH, mineralogy and redox conditions [2,3,4,34]. These properties can favour environmental mobility relative to many cationic trace metals while permitting substantial site-specific retention where suitable sorption mechanisms are present.

Tagami and Uchida [3] demonstrated that total soil Re does not necessarily represent the fraction potentially available for biological transfer. Although total concentrations were similarly low across the agricultural soils examined, the proportion recovered in water-soluble form varied substantially among soil types. Their subsequent nutrient-solution experiments showed comparable uptake and distribution behaviour for Re and Tc in radishes [4], providing direct evidence that chemically available Re can enter terrestrial biological systems.

Experiments conducted under deliberately Re-enriched conditions provide further evidence of biological accessibility. Tzvetkova et al. [5] reported substantial accumulation of Re in plant tissues together with high water extractability of leaf-associated Re. Direct imaging studies have visualised Re within exposed plant tissues [6], while recent experiments with *Lactuca sativa* demonstrated concentration-dependent uptake accompanied by physiological effects at elevated experimental exposures [7]. These studies establish uptake potential but do not provide transfer coefficients that can be applied quantitatively to wild fungi.

Element acquisition by fungi occurs within a biological and spatial framework distinct from that of vascular plants. Fruiting-body elemental concentrations may depend on fungal identity and physiology, trophic or ecological strategy, substrate composition, soil physicochemical properties, element-specific bioavailability and the microscale distribution of the underlying mycelium. Accordingly, paired bulk-soil or substrate concentrations may be only weak-to-moderate predictors of concentrations in fungal fruiting bodies [8,9,10]. Transfer relationships derived from vascular-plant systems therefore cannot be applied directly to mushrooms.

The site-level topsoil comparison provides useful environmental context, but the apparent contrast between soil and fungal Re must be interpreted in relation to the markedly different analytical resolution of the two matrices. All 52 topsoil subsamples representing the 26 site-level composites were below the operational soil Re threshold (approximately 79 ng g^−1^ dw), including the composites corresponding to the nine mushroom-collection sites eligible for site-level matching. By contrast, mushroom-associated Re signals occurred at four of these nine matched sites. However, the soil threshold was substantially higher than the concentration range relevant to much of the fungal dataset: the model-based mushroom median was 1.055 ng g^−1^ dw, and most fully quantified mushroom concentrations were also below the soil-method detection threshold. Moreover, previously reported Re concentrations in non-enriched agricultural soils are approximately 0.23–0.34 ng g^−1^ [3], more than two orders of magnitude below the present soil analytical threshold. Soil non-detection therefore cannot be interpreted as evidence that Re was absent from the park soils or that fungal Re necessarily originated from a non-soil source. Instead, the observations are compatible with uptake from low-level or chemically available soil pools below the analytical resolution of the bulk-soil method, microscale heterogeneity not captured by park-level composites, and acquisition across the spatial extent of fungal mycelia.

Atmospheric deposition nevertheless remains a plausible additional pathway. Re has previously been quantified in wild mushrooms collected near a heavily trafficked road [11], although that study did not establish a traffic-derived Re source. No Re-specific measurements of local particulate matter, wet or dry atmospheric deposition, or road dust were available for the present sampling area. Accordingly, no source attribution to traffic emissions or catalytic converters is made. Distinguishing among substrate-mediated and atmospheric pathways will require more sensitive Re determination in spatially paired substrates together with measurements of bioavailable Re fractions and atmospheric particulate or deposition inputs.

### 3.3. Human-Health Interpretation of Environmental Fungal Re

The detection of Re in fungal fruiting bodies establishes environmental occurrence but does not, by itself, quantify dietary exposure. Potential exposure would additionally depend on whether the relevant taxa are consumed, the amount and frequency of consumption, fresh- versus dry-weight relationships, and the effects of processing and cooking. More fundamentally, measurement of total elemental Re does not identify the chemical species released from the fungal matrix during gastrointestinal digestion or the fraction subsequently available for absorption.

Studies of trace elements in edible mushrooms have demonstrated that chemical speciation and gastrointestinal bioaccessibility can materially alter the fraction potentially available for systemic uptake and therefore the interpretation of exposure or risk estimates based solely on total elemental concentrations [18,19,20]. This distinction is particularly important for Re, because the toxicological evidence relevant to chronic environmental or dietary exposure is limited and strongly dependent on chemical form.

Early experimental studies reported comparatively low acute toxicity for soluble perrhenate salts, whereas other Re compounds exhibited substantially different toxicological behaviour [1,15,16]. A dedicated review of Re speciation and toxicity similarly concluded that the available toxicological database cannot be generalised across inorganic Re salts, organometallic compounds and coordination complexes [16]. Low acute toxicity of a particular perrhenate salt should therefore not be interpreted as evidence of the absence of biological activity or chronic hazard.

Perrhenate is biologically transportable. Zuckier et al. [17] demonstrated uptake through the sodium–iodide symporter (NIS) and tissue-specific biodistribution in vivo, including uptake by the thyroid, stomach and salivary glands. Contemporary toxicological summaries also characterise the evidence base for Re as limited while reporting thyroid effects following repeated high-dose ammonium perrhenate exposure in experimental animals [1]. These observations establish biological handling of particular Re species, but they do not provide a chronic oral dose–response relationship applicable to total Re measured in environmental mushrooms.

The present dataset therefore supports further exposure characterisation rather than quantitative health-risk estimation. A defensible dietary assessment would require consumption-relevant concentration data, the identification of environmentally relevant Re species, information on the effects of food processing, gastrointestinal bioaccessibility, systemic absorption and toxicokinetics and a chronic oral toxicological reference point applicable to the relevant chemical form [16,18,19,20,21]. These elements are not currently established for environmental fungal Re.

Accordingly, the concentrations reported here should be interpreted neither as evidence of adverse human-health risk nor as evidence of safety. Rather, they define an environmental-occurrence dataset and identify the additional evidence required to progress from external occurrence to biologically relevant dose and quantitative risk characterisation.

### 3.4. Literature Context: Environmental Re Versus Synthetic Re Compounds

This section provides external literature context rather than direct interpretation of the measured mushroom Re concentrations, and therefore distinguishes among digestion-based total elemental Re, environmentally plausible Re species and deliberately synthesised Re compounds. The ICP–MS approach used here quantified total elemental Re and did not preserve or resolve oxidation state, ligand environment or molecular speciation. Under oxic environmental conditions, Re(VII) may occur as the relatively soluble perrhenate oxyanion (ReO_4_^−^), which has demonstrated biological transport through the sodium–iodide symporter and tissue-specific biodistribution in experimental systems [16,17]. This evidence establishes that a chemically defined inorganic Re species can be biologically transported, but it neither demonstrates that Re detected in the present mushroom samples was present as perrhenate nor provides a toxicological benchmark for the measured total-Re concentrations.

A separate mechanistic literature concerns deliberately synthesised Re-containing compounds, including Re(I) tricarbonyl complexes, Re clusters and related coordination compounds developed primarily for biomedical applications [22,23,24,25,26,27,28,29,30,31,32,33]. These studies demonstrate that defined Re species can engage diverse cellular pathways and that biological activity depends strongly on chemical form, molecular structure, cellular uptake and subcellular localisation [16,22,23,24,25,26,27,28,29,30,31,32,33]. However, these pharmacological and compound-specific findings provide only external mechanistic context and hypothesis-generating evidence. They cannot be extrapolated quantitatively to ultratrace total elemental Re measured in environmental mushroom samples. The distinction among these evidence domains is summarised in Table 4. A detailed compound-by-compound summary of the mechanistic literature and its relevance to the interpretation of environmental Re is provided in Supplementary Table S8.

Accordingly, no molecular effect, toxicological response, human-health risk or safety conclusion can be inferred from the Re concentrations reported in the present study. Establishing a biologically relevant connection between environmental fungal Re and molecular toxicology would require identifying environmentally relevant Re species, assessing food-processing stability and gastrointestinal bioaccessibility, characterising of systemic absorption and internal dose, and experimentally investigating using chemical forms and exposure levels representative of realistic environmental or dietary exposure. The present findings should therefore be interpreted as environmental-occurrence data that define priorities for subsequent speciation and exposure-oriented research.

**Table 4:** Summary of Re evidence domains and their relevance to the present study.

Re Form/Evidence Domain	Evidence Base	Relevance to the Present Study
Total elemental Re (present study)	Digestion-based ICP–MS; chemical speciation not resolved	Directly measured; supports environmental-occurrence and censoring-aware concentration analysis
Re(VII) perrhenate (ReO_4_^−^)	Environmentally plausible soluble oxidised Re species; experimental evidence of biological transport [16,17]	Relevant to environmental mobility and biological-transport context, but not identified directly in the mushroom samples
Synthetic Re(I) complexes and Re clusters	Biomedical and pharmacological mechanistic studies [22,23,24,25,26,27,28,29,30,31,32,33]	Mechanistic and hypothesis-generating evidence only; no evidence that these compounds occur in the mushroom samples and not applicable to concentration-based risk inference

### 3.5. Analytical Interpretation of Ultratrace Re

The present dataset also illustrates the analytical consequences of extensive censoring in environmental ultratrace measurements. Only 20/121 fruiting-body units yielded exact reconstructed concentrations; more than four fifths of the dataset was represented by left- or interval-censored information.

Excluding censored observations would preferentially retain higher-concentration samples and distort the apparent concentration distribution. Conversely, substituting zero, one-half of the LLOQ, LLOQ/√2 or another fixed value would impose numerical concentrations that are not contained in the analytical evidence and could bias estimates of location and variability [35].

The primary analysis therefore preserved the information carried by each analytical state. Non-detects entered the analysis as left-censored observations bounded by their applicable analytical limits, detected-below-operational-LLOQ and partially quantified fruiting-body units entered as concentration intervals, and fully quantified units entered as exact values. This representation allowed the available information from all 121 biological units to contribute to estimation of the underlying concentration distribution without replacing censored observations with arbitrary surrogate concentrations.

The contrast between the median of the 20 exact observations (10.562 ng g^−1^ dw) and the interval-censored model-based median for the complete dataset (1.055 ng g^−1^ dw) illustrates the magnitude of complete-case selection in a dataset containing 83.5% non-exact observations. The exact-subset median should therefore be interpreted as a descriptive summary of the fully quantified portion of the distribution rather than as an estimate of the population median.

Importantly, the population-level estimate was not materially dependent on the primary lognormal assumption among the best-supported candidate distributions. Lognormal and log-logistic models showed essentially equivalent information-criterion support and yielded nearly identical medians. This distributional sensitivity analysis strengthens confidence that the principal estimate is not an artefact of selecting a single parametric form. The poorer fit and lower medians obtained with Weibull and gamma models also illustrate why the underlying distributional assumption should be evaluated explicitly in heavily censored ultratrace datasets.

The interval-censored likelihood was particularly appropriate because the reconstructed dataset contained not only left-censored observations but also substantial interval censoring arising from the cap–stem reconstruction. Methods developed primarily for left-censored datasets, including regression on order statistics, can provide useful sensitivity information but do not represent this interval structure as completely as the likelihood-based approach used for the primary analysis [35,36].

Analytical background represented a second major determinant of classification because many Re responses occurred close to background variability. Sensitivity analyses were therefore used to quantify the dependence of occurrence classification on alternative defensible background definitions rather than treating any single background specification as analytically invariant.

Inclusion of the earlier elevated procedural-blank phase in Scenario C markedly reduced the number of above-threshold classifications, demonstrating the sensitivity of ultratrace occurrence estimates to procedural-background definition. By contrast, classification remained identical for all 59 fruiting-body units directly comparable under Scenario A and the stricter same-batch Scenario B. Thus, although background specification materially affects the conservative boundary of interpretation, application of the stricter same-batch criterion did not alter any classification within the subset for which that comparison could be performed.

### 3.6. Strengths and Limitations

A principal strength of the study is the explicit reconstruction of the biological unit of analysis. Separately analysed cap and stem portions were not treated as independent fruiting bodies. Instead, 157 original analytical records were reconciled to 153 included analytical portions and ultimately to 121 fruiting-body-level biological units. This approach avoided pseudoreplication while retaining the analytical information contained in separately analysed tissues.

Additional strengths include retention of individual analytical-portion masses, derivation of sample-specific dry-weight analytical limits, explicit separation of detection from quantification, preservation of left- and interval-censored information, sensitivity analysis of alternative analytical-background definitions, bootstrap estimation of uncertainty and maintenance of a traceable analytical workflow. Together, these features are particularly important for an ultratrace dataset in which analytical uncertainty constitutes a substantial component of the observed concentration structure.

Several limitations constrain biological and toxicological interpretation. First, mushroom sampling was opportunistic and unbalanced among taxa and locations, and collection location, taxonomic status and analytical batch were not independently distributed. Castle Garden provides the clearest example: all 17 fruiting-body units from this location belonged to the ITS-unresolved subset, all were analysed within batches B01–B02, and the site contributed 14 of the 20 fully quantified observations. Location, taxonomic status and analytical batch are therefore structurally confounded for this site, preventing attribution of the Castle Garden Re profile to an independent environmental-location effect. The corresponding site-specific pattern should be regarded as descriptive and hypothesis-generating only. Moreover, the genotypic identity and spatial extent of the underlying mycelia were not determined, so multiple fruiting bodies collected within the same location cannot necessarily be assumed to represent independent fungal genets.

The sampling design also does not support inference to UK green spaces or to UK fungal diversity more generally. Collections reflected fruiting bodies available at the selected sites during 12 sampling dates between September and November 2019 and therefore represent a temporally restricted convenience sample rather than a seasonally or interannually replicated survey. Consequently, the observed occurrence frequencies and concentration distribution should not be interpreted as population-level estimates for wild mushrooms across the United Kingdom or across broader fungal communities.

Second, the study quantified digestion-based total elemental Re rather than individual Re chemical species. Oxidation state, molecular speciation, intracellular or subcellular distribution, gastrointestinal bioaccessibility and systemic bioavailability were therefore outside the analytical scope. This limitation is particularly important for toxicological interpretation because existing evidence demonstrates the pronounced chemical-form dependence of Re biological behaviour.

Third, the associated topsoil composites represented park-scale geochemical conditions rather than microscale substrates spatially paired with individual fruiting bodies, and numerically resolved site-level Re concentrations were unavailable. Quantitative soil–fungus transfer could therefore not be evaluated.

Fourth, none of the available matrix reference materials carried an assigned Re concentration. Consequently, a Re-specific matrix recovery or trueness estimate could not be derived. The botanical reference material and other quality-control procedures support the broader digestion and instrumental workflow, but they should not be interpreted as direct validation of Re-specific matrix recovery.

Fifth, the estimated population median is conditional on the fitted lognormal interval-censored model. Bootstrap resampling quantified numerical uncertainty within that modelling framework, and all bootstrap fits converged, but this does not establish that the lognormal distribution is uniquely correct. Lower matrix-specific analytical limits and larger datasets containing a greater proportion of exact observations would permit stronger evaluation of the underlying concentration distribution and alternative distributional assumptions.

Finally, identification of the post-replacement procedural-blank population relied on documented laboratory history rather than a contemporaneously dated record of the exact procedural change. Earlier blank records were therefore retained in the analytical archive and their influence was evaluated explicitly through the conservative sensitivity analysis rather than being removed from consideration. This treatment preserves transparency regarding analytical background while allowing the consequences of alternative background definitions to be quantified.

## 4. Materials and Methods

### 4.1. Study Area and Mushroom Sampling

Wild mushroom fruiting bodies were collected from public green spaces in Leicester and selected rural locations in Leicestershire, East Midlands, United Kingdom, as part of a broader environmental-biomonitoring programme.

Field sampling was conducted on 12 dates between 12 September and 18 November 2019. Collections were undertaken under dry conditions following approximately 24 h without rainfall, with recorded ambient temperatures ranging from approximately 8 to 17 °C. Fruiting bodies occurring naturally within accessible areas of the selected green spaces were located visually, and their geographical positions were recorded using a handheld Global Positioning System (GPS) device.

Only fresh, young and macroscopically intact fruiting bodies were selected. Specimens showing substantial decomposition, insect damage, visible parasitism, trampling or other marked deterioration were excluded, as were fruiting bodies growing in conspicuously littered or heavily disturbed microsites.

Nitrile gloves were worn during collection to minimise external contamination and direct contact with potentially toxic or inedible taxa. Complete fruiting bodies were collected where practicable, placed individually in labelled sealed polypropylene bags and transported to the laboratory for taxonomic assessment and trace-element preparation.

Sampling reflected the natural availability of fruiting bodies and was not designed as a balanced taxonomic experiment. No minimum separation distance was imposed between specimens, and neither the genotypic identity nor the spatial extent of the underlying mycelia was determined. Multiple fruiting bodies collected from the same location should therefore not necessarily be regarded as independent fungal genets.

### 4.2. Taxonomic Identification and Molecular DNA Barcoding

Taxonomic identification was based initially on the macroscopic characteristics of the collected fruiting bodies and was subsequently confirmed by molecular DNA barcoding using the fungal ribosomal internal transcribed spacer (ITS) region.

For molecular identification, approximately 100 mg of frozen mushroom material was cryogenically homogenised with liquid nitrogen using a ceramic mortar and pestle and transferred to a microcentrifuge tube. Genomic DNA was extracted using the DNeasy Plant Mini Kit^®^ (QIAGEN Inc., Germantown, MD, USA) according to the manufacturer’s instructions and a previously published DNA-barcoding workflow [37]. DNA concentration and purity were assessed spectrophotometrically from the A260/A280 absorbance ratio using a NanoDrop Lite Spectrophotometer (Thermo Scientific, Madison, WI, USA; instrument reference 269-275200). An A260/A280 ratio of approximately 1.7–2.0 was considered indicative of DNA of sufficient purity for subsequent PCR amplification.

The fungal ITS region was amplified using the universal ITS1 and ITS4 primers originally described for fungal nrDNA amplification [38], consistent with the established use of ITS as the principal universal DNA barcode for fungi [39]. The conventional nrITS amplification, gel-electrophoresis and sequencing workflow was based on the laboratory protocol previously described by Sgamma et al. [37]. The primer sequences were ITS1, 5′-TCCGTAGGTGAACCTGCGG-3′, and ITS4, 5′-TCCTCCGCTTATTGATATGC-3′. PCR was performed in a final reaction volume of 10 µL containing 5 µL of MyTaq polymerase preparation (Bioline, London, UK), 0.1 µL of forward ITS1 primer (0.2 µM), 0.1 µL of reverse ITS4 primer (0.2 µM), template DNA and nuclease-free water to the final reaction volume. Amplification was carried out using a PTC-200 Peltier Thermal Cycler (MJ Research Inc., Waltham, MA, USA). The thermal programme comprised an initial denaturation at 94 °C for 2 min, followed by 40 cycles of denaturation at 94 °C for 15 s, annealing at 60 °C for 30 s and extension at 72 °C for 30 s, followed by holding at 4 °C.

PCR products were evaluated by agarose-gel electrophoresis. A 2% (*w*/*v*) agarose gel was prepared in electrophoresis buffer using molecular-biology-grade agarose (Sigma-Aldrich, St. Louis, MO, USA; product A9539) and SYBR™ Safe DNA Gel Stain (Invitrogen, Paisley, UK). EasyLadder I (Bioline, UK) was included as a molecular-size marker. Electrophoresis was performed at 100 V for approximately 30 min, and amplified DNA products were visualised using a Gel Doc™ EZ gel documentation system (Bio-Rad, Hercules, CA, USA).

Amplicons selected for sequencing were excised from the agarose gel and purified using the documented gel-solubilisation/Sephaglass procedure. Briefly, gel fragments were treated with gel solubiliser and incubated at 60 °C, followed by binding to resuspended Sephaglass beads, centrifugation, repeated washing and final elution of the purified PCR product. The purified amplicons were then transferred to fresh microcentrifuge tubes for sequence analysis.

Purified PCR products were submitted to Macrogen Europe for Sanger sequencing. The resulting sequence reads were assembled into consensus ITS sequences using CLC Main Workbench version 7.5.1 (QIAGEN, Germantown, MD, USA). Consensus sequences were subsequently interrogated against reference nucleotide sequences using the asic Local Alignment Search Tool (BLAST version 2.13.0; National Center for Biotechnology Information, National Library of Medicine, Bethesda, MD, USA; accessed in 2022) [40] to support species-level taxonomic assignment.

At the reconstructed fruiting-body level, successful ITS amplification and interpretable sequence information were obtained for 104/121 units (86.0%), corresponding to all specimens assigned to a named taxon. For the remaining 17/121 units (14.0%), genomic DNA was extracted but repeated PCR attempts did not yield an ITS amplicon suitable for sequencing. These specimens were therefore retained as unknown/unidentified taxa in the complete analytical dataset but were excluded from named-taxon biological interpretation. The cause of unsuccessful amplification was not investigated experimentally; therefore, no specific mechanism was assigned to these failures. These 17 units corresponded to all 17 fruiting bodies collected at Castle Garden; thus, their unresolved taxonomic status resulted from unsuccessful ITS amplification rather than from ambiguous species-level BLAST assignment.

No predefined minimum BLAST percentage-identity or query-coverage threshold was available in the molecular records. Taxonomic identities therefore correspond to the original ITS-supported assignments, and no new numerical sequence-matching threshold was imposed for the present analysis.

### 4.3. Cleaning, Tissue Separation and Homogenisation

In the laboratory, visible debris, insects and adherent soil particles were removed manually from the fruiting-body surfaces. Samples were washed with tap water, rinsed with deionised water and finally rinsed with Milli-Q^®^ ultrapure water produced using a Milli-Q^®^ Direct 8 purification system (Merck Millipore, Darmstadt, Germany; resistivity 18.2 MΩ·cm).

Where sufficient biomass was available, fruiting bodies were separated into cap and stem fractions before drying to preserve tissue-specific analytical information. Specimens with insufficient material for tissue separation were processed as whole fruiting bodies.

Cleaned mushroom material was oven-dried at 50 °C until constant weight, typically overnight. The dried material was cryogenically homogenised with liquid nitrogen using a ceramic mortar and pestle until a fine, visually homogeneous powder was obtained. No sieving step was applied to the mushroom material. Homogenised samples were thoroughly mixed, transferred to pre-cleaned capped polypropylene tubes and stored at −80 °C until digestion.

Reusable sample-contact equipment, including forceps, spatulas, mortars and pestles, was cleaned between specimens using laboratory detergent where appropriate, followed by deionised water, 5% (*v*/*v*) HNO_3_ and repeated rinsing with Milli-Q ultrapure water. Glassware was avoided where practicable during trace-element preparation. The broader mushroom-sample preparation workflow has been described previously for the associated Leicester multielement programme [41].

### 4.4. Acid Digestion and Concentration Reconstruction

Dried, homogenised mushroom material was subjected to closed-vessel, non-microwave acid digestion in pre-cleaned Teflon vessels. Where sufficient biomass was available, approximately 0.500 g dry weight of homogenised material was accurately weighed into each vessel. Smaller analytical portions were retained where sample availability was limited, and the exact dry mass of each analytical portion was incorporated individually into all dry-weight concentration and analytical-limit calculations.

Five millilitres of 65% (*v*/*v*) HNO_3_ were added to each analytical portion, followed by 1 mL of 30% (*v*/*v*) H_2_O_2_; both reagents were Suprapur^®^ grade (Merck KGaA, Darmstadt, Germany). The sealed vessels were maintained at room temperature for approximately 8 h to allow pre-digestion and were subsequently heated at 96 °C for 12 h.

After cooling, digestates were filtered through Whatman™ Grade 42 ashless quantitative filter paper (Cytiva, Marlborough, MA, USA) and quantitatively diluted to a final volume of 25 mL with Milli-Q ultrapure water. Before ICP–MS analysis, an aliquot of each digest was additionally passed through a 0.45 µm PTFE syringe filter to remove residual particulate material from the instrumental sample-introduction pathway.

Immediately before ICP–MS measurement, mushroom digestates were manually diluted 11-fold. The automated prepFAST sample-introduction system did not apply an additional dilution to these analytical records. Accordingly, the ×11 pre-instrument dilution factor was applied once during concentration reconstruction.

Dry-weight Re concentrations were calculated according to:

*C*_Re_ (ng g^−1^ dw) = *C*_introduced_ (µg L^−1^) × 11 × 25/*m*

where *C*_introduced_ is the Re concentration in the solution introduced into the ICP–MS and *m* is the dry mass of the analysed mushroom portion in grams. The same conversion was applied to the solution-scale operational detection threshold and operational LLOQ, producing analytical-portion-specific dry-weight limits. The concentrations reported therefore represent digestion-based elemental Re recovered in the filtered mushroom digest and do not provide information on Re oxidation state, molecular speciation or subcellular localisation.

### 4.5. ICP–MS Acquisition, Calibration and Quality Assurance

Rhenium determination was performed at the Geography Research and Teaching Laboratories (Geolabs), School of Geography and the Environment, University of Oxford, Oxford, UK. ^187^Re was measured using a NexION 2000B inductively coupled plasma mass spectrometer (PerkinElmer Inc., Waltham, MA, USA) coupled to a prepFAST M5 automated sample-introduction system (Elemental Scientific Inc., Omaha, NE, USA). Instrument control and data acquisition were performed using Syngistix™ Software for ICP–MS, version 3.2 (PerkinElmer Inc., Waltham, MA, USA). The broader Oxford analytical workflow used for trace and ultratrace elements in these mushroom digestates has been described for the associated analytical programme [41].

The multielement acquisition protocol used peak hopping with 20 sweeps per reading, one reading per replicate and five instrumental replicates per analytical record. ^187^Re was acquired in standard mode without collision-cell gas, using a dwell time of 25 ms per atomic mass unit and a total integration time of 500 ms. The instrumental acquisition method applied a mathematical interference correction of −0.121660 × the ^189^Os response to the ^187^Re signal. Identical Re acquisition settings were applied to calibration standards, blanks, quality-control materials and mushroom digestates.

Sample-specific instrumental precision was evaluated from the archived Syngistix ^187^Re relative standard deviation (%RSD) associated with the five instrumental replicate measurements for each analytical record. For fully quantified units represented by a single analytical record, the corresponding instrumental standard deviation (SD) in ng g^−1^ dw was derived as the reconstructed dry-weight concentration × RSD/100. Because dry-weight conversion is a linear scaling operation, the relative standard deviation is unchanged by this transformation. For the two fully quantified units reconstructed from separately analysed cap and stem components, component-specific instrumental SDs were propagated through the dry-mass-weighted reconstruction according to $SD_{unit}=\sqrt{\left(w_{cap}SD_{cap}{)}^{2}+(w_{stem}SD_{stem}{)}^{2}\right.}$, where w denotes the recorded dry-mass weighting fraction. These precision estimates represent within-run instrumental replicate variability and not total procedural or method uncertainty.

External calibration for ^187^Re comprised five non-zero standards at 0.005, 0.011, 0.027, 0.055 and 0.137 µg L^−1^. Calibration standards and independently sourced quality-control materials were prepared from separate commercial reference solutions used by the Oxford analytical facility. Linear regression across the calibration interval yielded *R*^2^ = 0.999476. The lowest calibration standard, 0.005 µg L^−1^, showed an RSD of 7.15% and a back-calculation error of −14.05% and was therefore retained as the study-specific operational LLOQ. The designation *operational* is used because the available archived evidence does not constitute a complete matrix-specific metrological validation of the quantification limit. The calibration data supporting the selection of the operational LLOQ are reported in Table S1.

Thus, satisfactory overall calibration linearity was not taken to imply equivalent quantitative performance at the lower end of the calibration range. Instead, low-end quantitative performance was evaluated from the observed RSD and back-calculation error of the lowest calibrator, supporting its use as a study-specific operational LLOQ rather than as a formally validated matrix-specific LoQ. The background-derived 3 × SD criterion was used separately to define the operational detection threshold and was not added to the lowest calibration standard when defining the operational LLOQ, because detection and quantification were treated as distinct analytical decision levels. At 0.005 µg L^−1^, the observed RSD (7.15%) and back-calculation error (−14.05%) were both within ±20% numerical bounds commonly used as a practical lower-limit performance criterion; however, this does not constitute regulatory-level or matrix-specific LoQ validation. The negative back-calculation error at the lowest calibrator indicates greater relative uncertainty and a potential tendency toward underestimation for sample responses close to the lower calibration boundary; it was not applied as a correction factor because it does not represent a validated sample- or matrix-specific bias estimate.

Quality assurance incorporated complementary controls addressing different components of analytical performance: procedural acid-digestion blanks, instrumental 2% HNO_3_ washes, ^115^In internal-standard normalisation, calibration-series performance, an independently sourced aqueous Re quality-control solution and a botanical matrix reference material. Each control was interpreted only for the analytical-performance characteristic that it directly assessed.

NIST SRM 1570a Trace Elements in Spinach Leaves (National Institute of Standards and Technology, Gaithersburg, MD, USA) was processed alongside the mushroom samples as a botanical-matrix reference material. SRM 1570a was not selected as a Re-specific validation material. Rather, it was included because Re determination formed part of a broader multielement botanical analytical programme and therefore served as a botanical-matrix processing control for elements with assigned values. The certificate for SRM 1570a does not provide a certified, reference or information mass-fraction value for Re. Consequently, a Re-specific mushroom-matrix recovery could not be calculated. For elements possessing assigned values, recoveries obtained within the same broader mushroom analytical programme ranged from 79.8% to 120.2% [41]. These recoveries support the general digestion and analytical workflow in a botanical matrix but do not constitute validation of Re-specific recovery or trueness.

An independently sourced high-concentration aqueous Re reference solution was measured in triplicate and produced an RSD of 3.03%, supporting high-level instrumental repeatability. Because this solution did not undergo the mushroom digestion procedure and was analysed outside the calibration interval used for the environmental samples, it was not used to estimate matrix recovery, within-range accuracy or analytical trueness.

^115^In was used as the internal standard and was automatically introduced by the prepFAST M5 system at a nominal concentration of 1 ng g^−1^ into calibration standards, blanks and samples. The analytical records confirmed consistent internal-standard introduction across the analytical sequence and use of the standard-mode ^115^In response for analytes acquired under standard conditions, including ^187^Re. Internal-standard responses were used to monitor and normalise instrumental and matrix-related signal variation and were not treated as a surrogate for Re-specific matrix recovery.

The analytical sequence included initial 2% HNO_3_ wash measurements, calibration standards, independent quality-control material, a subsequent wash and the mushroom samples, with additional washes interspersed throughout the sequence and repeat quality-control measurements later in the run. Between measurements, the prepFAST M5 system rinsed and refilled the injection loop with clean 2% HNO_3_ to minimise carry-over. Wash measurements were retained as instrumental-background diagnostics and were not treated as procedural digestion blanks.

Eighteen acid-digestion blank records were available for the Re analytical reconstruction. Eight blanks (IDs 121, 122, 140, 141, 159, 160, 178 and 179) belonged to the procedural phase following replacement of a Teflon vessel associated with the earlier elevated Re blank responses. These eight blanks defined the primary procedural-background population used for Scenario A. The earlier blank records were included only in the deliberately conservative Scenario C sensitivity analysis.

Within the eight-blank Scenario A population, the mean and median signed Re responses were −0.000711 and −0.000720 µg L^−1^, respectively. The conventional standard deviation was 0.000224 µg L^−1^ and the robust standard deviation was approximately 0.000223 µg L^−1^. Signed analytical responses were preserved during reconstruction and were not interpreted as negative environmental concentrations.

The instrumental wash measurements used to characterise wash variability had an SD of approximately 0.000349 µg L^−1^. For Scenario A, the background-location term was constrained to the non-negative median of the post-replacement procedural blanks and was therefore zero. The operational detection threshold was defined as:

*T* = max(3 SD_wash_, 3 SD_blank,robust_)

This criterion yielded a Scenario A operational detection threshold of 0.001046 µg L^−1^ on the introduced-solution scale, with wash variability determining the threshold. The maximum operator was used deliberately to ensure that the operational threshold was not set below the larger of the instrumental-wash or procedural-blank variability terms, thereby providing a conservative safeguard against underestimating background fluctuation at ultratrace signal levels. Conversion of both the operational detection threshold and operational LLOQ to dry-weight units incorporated the exact analytical-portion mass, the 25 mL final digest volume and the ×11 pre-instrument dilution, thereby producing sample-specific analytical limits.

Overall, the QA/QC framework documented calibration linearity, replicate instrumental precision, internal-standard normalisation and control of instrumental and procedural background. Because no available botanical matrix reference material carried an assigned Re concentration, no Re-specific matrix recovery percentage is reported.

### 4.6. Fruiting-Body Reconstruction and Analytical Classification

The original analytical dataset comprised 157 mushroom analytical records, including whole-fruiting-body measurements and separately analysed cap and stem portions. Four unmatched tissue-only records could not be reconstructed into complete fruiting-body units and were excluded from the principal biological dataset. The remaining 153 analytical portions comprised 89 whole-fruiting-body records and 64 tissue-specific records corresponding to 32 matched cap–stem pairs. Reconstruction therefore yielded 121 fruiting-body-level biological units.

At analytical-portion level, Scenario A observations were assigned to mutually exclusive analytical categories using the background-adjusted Re response and the study-specific operational limits. Responses at or below the operational detection threshold were classified as non-detects (ND). Responses exceeding the operational detection threshold but remaining below 0.005 µg L^−1^ were classified as detected below the operational LLOQ. Responses from 0.005 to 0.137 µg L^−1^, inclusive, were considered quantitatively valid within the calibration interval. Responses exceeding the upper calibration level of 0.137 µg L^−1^ were classified as above the calibration range. Observations lacking sufficient analytical information for valid classification under a particular sensitivity scenario were designated not evaluable.

Whole-fruiting-body analytical portions were retained directly as individual biological units. For the 32 matched cap–stem pairs, concentrations or analytical bounds were reconstructed separately for each component. Fruiting-body-level lower and upper bounds were then obtained by weighting component values according to the recorded dry masses of the analysed portions. Dry-mass weighting was used because the cap and stem analytical portions contributed unequal recorded masses to the reconstructed analysed material; simple averaging would instead assign equal influence to both components irrespective of the amount of material represented.

Pairs for which both components were quantitatively valid yielded an exact reconstructed fruiting-body concentration and were classified as fully quantified. Units containing at least one quantitatively valid component but not supporting an exact reconstructed fruiting-body concentration were classified as partially quantified. Units containing no quantified component but at least one response above the operational detection threshold were classified as detected below the operational LLOQ. All remaining evaluable fruiting-body units were classified as ND.

The recorded cap and stem dry masses represented the quantities of analytical material introduced into digestion and were used solely for fruiting-body-level concentration reconstruction. They should not be interpreted as experimentally determined whole-fruiting-body cap:stem biomass proportions. Eligibility of the 32 matched pairs for tissue-specific quantitative comparison is summarised in Table S5.

### 4.7. Analytical-Background Definition and Sensitivity Analyses

Because Re was measured at ultratrace concentrations close to instrumental and procedural background, the analytical-background definition was incorporated explicitly into the classification. Scenario A constituted the primary analytical interpretation; Scenarios B and C were retained as procedural-background sensitivity analyses, and Scenario D was used as an instrumental-background diagnostic.

Scenario A used the eight procedure-defined post-replacement acid-digestion blanks described in Section 4.5 and served as the primary background definition.

Scenario B imposed a stricter same-batch requirement. An analytical portion was evaluable only when an eligible post-replacement procedural blank was available from the same analytical batch. This criterion increased batch specificity but reduced the evaluable dataset because an eligible same-batch blank was not available for every analytical portion.

Scenario C incorporated all 18 archived acid-digestion blanks, including those generated during the earlier procedural phase characterised by elevated blank responses. Scenario C was deliberately retained as a conservative sensitivity boundary and was not interpreted as an alternative estimate of the primary Re concentration distribution.

Scenario D used wash-derived instrumental background as a diagnostic comparator. Because instrumental washes do not reproduce the mushroom digestion procedure, Scenario D was used only to assess the influence of instrumental background and was not considered an independent procedural-background definition.

Agreement among analytical-background scenarios was evaluated at the fruiting-body level. Comparisons involving Scenario B were restricted to fruiting-body units for which the stricter same-batch criterion could be applied. The complete classification counts across Scenarios A–D are provided in Table S6.

Analytical batches were coded B01–B10 for the batch-based sensitivity analyses.

### 4.8. Statistical Analysis

Analytical occurrence was summarised at the fruiting-body level using counts and proportions. Exact binomial 95% confidence intervals for the four Scenario A occurrence categories were calculated using the Clopper–Pearson method [42].

The primary concentration analysis preserved the censoring structure generated by analytical classification. Non-detects were represented as left-censored observations, fruiting-body units constrained by lower and upper analytical bounds as interval-censored observations, and fully quantified units as exact concentrations. This approach follows established recommendations for censored environmental datasets, for which replacement of censored observations by zero or fixed fractions of detection or quantification limits can bias estimates of distributional location and variability [35].

Because 101/121 Scenario A fruiting-body observations (83.5%) were non-exact, the principal distributional analysis used an intercept-only parametric interval-censored model. The intercept-only specification was deliberately retained for population-level estimation because the opportunistic sampling design, extensive censoring and strong confounding among collection location, taxonomic status and analytical batch did not support stable and independently interpretable covariate-adjusted effects. A lognormal distribution was retained as the primary parametric form and was fitted using Surv(..., type = “interval2”) and survreg from the R survival package [36]. The interval-censored likelihood allowed exact, left-censored and interval-censored observations to contribute jointly without fixed-value substitution [35,36].

Because distributional assumptions may exert substantial influence when censoring is extensive, a distributional sensitivity analysis was additionally performed using exactly the same 121 fruiting-body units and the same lower and upper analytical bounds as in the primary analysis. Intercept-only interval-censored maximum-likelihood models assuming lognormal, log-logistic, Weibull and gamma distributions were compared using the Akaike information criterion (AIC) and Bayesian information criterion (BIC), and the corresponding model-based medians were compared. All four alternative distributional fits converged successfully. The fitted intercept on the logarithmic scale was exponentiated to obtain the model-based median Re concentration of the underlying distribution.

Uncertainty in the model-based median was evaluated by non-parametric bootstrap resampling at the fruiting-body level, thereby preserving the biological unit of analysis. A total of 1000 bootstrap datasets were generated by resampling complete fruiting-body units with replacement, and the interval-censored model was refitted to each resample. All 1000 bootstrap fits converged successfully. The empirical 2.5th and 97.5th percentiles of the resulting median estimates were used to construct the 95% bootstrap confidence interval [43].

Regression on order statistics (ROS), implemented using NADA::cenros, was retained as a secondary sensitivity method. ROS is widely used for left-censored environmental datasets [35]. However, the reconstructed fruiting-body dataset also contained substantial interval censoring. Because the primary interval-censored likelihood represented this mixed censoring structure more directly, ROS-derived summaries were not used as the principal distributional estimates.

No substitution of zero, LLOQ/2, LLOQ/√2, the LLOQ itself or any other fixed surrogate concentration was used in the primary concentration analysis [35].

Concordance among analytical-background scenarios was assessed at the fruiting-body level, with Scenario A–B agreement calculated only for units evaluable under both definitions. Location, area type, quadrant and taxonomic occurrence profiles were summarised descriptively rather than subjected to inferential group comparisons because the opportunistic sampling design produced an unbalanced representation of taxa, locations and analytical batches.

All statistical analyses were performed using R version 4.3.1 (R Foundation for Statistical Computing, Vienna, Austria) [44]. The principal analytical workflow used the survival, boot, NADA, NADA2, dplyr, tidyr, readr and ggplot2 packages. Package versions and reproducible analytical scripts were retained with the study outputs.

### 4.9. Contextual Topsoil Programme

The mushroom survey formed part of a broader Leicester and Leicestershire environmental-monitoring programme that also included surface topsoils collected from urban and rural green spaces [45]. Soil sampling targeted the upper 0–5 cm horizon. Across the wider programme, 850 individual field increments were pooled within sampling locations and homogenised to generate 26 site-level composite topsoils, which constituted the environmental units for spatial interpretation. Two separately identified physical subsamples were retained from each composite for the Re-related analytical programme, yielding 52 analytical records for laboratory QA/QC and sensitivity assessment while preserving *N* = 26 as the environmental sample size.

Composite topsoil aliquots in the broader analytical programme were subjected to microwave-assisted acid digestion using HNO_3_ and HCl, both Suprapur^®^ grade (Merck KGaA, Darmstadt, Germany), in a Multiwave GO microwave digestion system (Anton Paar GmbH, Graz, Austria). After cooling, digestates were filtered through Whatman™ Grade 42 ashless quantitative filter paper and diluted with Milli-Q ultrapure water. These soil procedures formed part of the companion Leicester/Leicestershire geochemical programme [41,45].

Quality control included CRM059-50G Trace Metals–Loamy Clay 2. Recoveries obtained for elements possessing assigned CRM values ranged from 94.9% to 108.6% [41], supporting the general soil digestion and instrumental workflow. CRM059 does not provide an assigned Re mass fraction and therefore could not be used to determine Re-specific soil recovery. For ^187^Re, the archived soil-data workflow applied a study-specific detection threshold of 0.0718 µg L^−1^ on the 11-fold-diluted analytical-solution scale, derived as three times the standard deviation of the instrumental wash measurements. This operational threshold was used for final below-DL classification.

The topsoil dataset was used to characterise park-scale geochemical conditions and for exploratory site-level contextual matching with the mushroom dataset. Mushroom and topsoil sites were considered eligible for matching when park names corresponded directly or through a previously validated site alias. Nine mushroom-collection sites met this criterion. Each matched site contributed one independent site-level soil composite. The two physical subsamples derived from each composite were retained as laboratory/preparation replications rather than treated as independent environmental soil samples.

The composite soils were not collected directly beneath individual fruiting bodies and therefore do not constitute microscale soil–fungus pairs. For ^187^Re, the archived soil-data workflow applied a study-specific operational detection threshold of 0.0718 µg L^−1^ on the 11-fold-diluted analytical-solution scale, derived as three times the standard deviation of the instrumental wash measurements. Under the documented soil-preparation conditions, this corresponded to approximately 79.0 ng g^−1^ dw. All 52 physical topsoil subsamples were below this threshold. Consequently, exact site-level soil Re concentrations, quantitative soil–mushroom correlations, concentration ratios, bioconcentration factors and transfer factors could not be calculated.

## 5. Conclusions

This study provides evidence of Re occurrence in an opportunistic sample of wild mushroom fruiting bodies collected from selected green spaces in Leicester and Leicestershire, United Kingdom. Under the primary analytical scenario, 48/121 fruiting-body units contained evidence of Re above the operational detection threshold, including 20 fully quantified and seven partially quantified units. Fully quantified concentrations ranged from 3.91 to 163.70 ng g^−1^ dw.

Preservation of the analytical censoring structure was essential, because 83.5% of the dataset consisted of non-exact observations. The interval-censored lognormal model estimated a population median Re concentration of 1.055 ng g^−1^ dw (95% bootstrap confidence interval: 0.672–1.571 ng g^−1^ dw), while complete classification concordance among all 59 units directly comparable under the primary and stricter same-batch procedural-background definitions supported the robustness of the principal occurrence classification.

These findings extend the limited evidence for Re occurrence in wild fungi outside environments selected for known Re mineralisation, but they should not be interpreted as representative of UK green spaces, broader fungal diversity, or seasonal and interannual variation. However, total elemental Re concentrations alone cannot define dietary exposure or human-health risk because chemical speciation, food-processing behaviour, gastrointestinal bioaccessibility, systemic absorption and internal dose remain unresolved. The present data should therefore be interpreted as evidence of environmental occurrence rather than as evidence of either adverse risk or safety. Future studies should combine taxonomically resolved fungal sampling with spatially paired substrate measurements, Re speciation and gastrointestinal bioaccessibility analyses to connect environmental occurrence with realistic exposure and toxicological dose–response relationships.

## Figures and Tables

**Table 1:** Summary of analytical performance and quality assurance for ^187^Re determination in wild mushrooms.

Parameter	Result
Monitored isotope	^187^Re
ICP–MS acquisition mode	Standard mode; no collision-cell gas
Peak-hopping acquisition	20 sweeps per reading; 1 reading per replicate; 5 instrumental replicates
Dwell time/integration time	25 ms/500 ms
Mathematical interference correction	^187^Re response corrected using −0.121660 × ^189^Os
Manual pre-ICP–MS dilution	×11, applied once
Final digest volume	25 mL
Calibration levels	0.005, 0.011, 0.027, 0.055 and 0.137 µg L^−1^
Calibration *R*^2^	0.999476
Lowest calibrator/operational LLOQ	0.005 µg L^−1^
RSD at lowest calibrator	7.15%
Back-calculation error at lowest calibrator	−14.05%
Primary procedural-blank population (Scenario A)	*n* = 8 (IDs 121, 122, 140, 141, 159, 160, 178, 179)
Scenario A operational detection threshold	0.001046 µg L^−1^ on the introduced-solution scale
Internal standard	^115^In, nominal 1 ng g^−1^; automatically introduced by prepFAST into calibration standards, blanks and samples
Independent aqueous Re reference	Triplicate analysis; RSD 3.03%; high-level instrumental repeatability check only
Botanical matrix reference material	NIST SRM 1570a Trace Elements in Spinach Leaves; no assigned Re value
Re-specific matrix recovery	Not calculable from the available matrix reference materials
Broader botanical CRM performance	Previously reported recovery range 79.8–120.2% for elements with assigned values (see Section 4.5)

The 0.005 µg L^−1^ value is a study-specific operational LLOQ and not a metrologically validated matrix-specific LoQ. NIST SRM 1570a supports the broader botanical-matrix workflow for elements with assigned values, but it does not provide an assigned Re concentration and therefore cannot establish Re-specific recovery or trueness.

**Table 2:** Re occurrence and censoring profile among 121 reconstructed wild-mushroom fruiting-body units under Scenario A. (**A**) Overall occurrence and censoring profile. (**B**) Individual fully and partially quantified fruiting-body units and archived instrumental precision.

(A)							
Classification			*N*	*n*	%	Exact 95% CI (%)	
Non-detect			121	73	60.3	51.0–69.1	
Detected below operational LLOQ			121	21	17.4	11.1–25.3	
Partially quantified			121	7	5.8	2.4–11.6	
Fully quantified			121	20	16.5	10.4–24.4	
(B)							
Unit	Final Taxon	Park	Classification	Re Result (ng g^−1^ dw)	Instrumental SD (ng g^−1^ dw)	Archived ^187^Re RSD (%)	Batch
FB001	Unknown/unidentified taxon	Castle Garden	Fully quantified	9.786	12.602	128.77	B01
FB002	Unknown/unidentified taxon	Castle Garden	Fully quantified	12.657	13.558	107.12	B01
FB003	Unknown/unidentified taxon	Castle Garden	Fully quantified	4.122	3.488	84.62	B01
FB004	Unknown/unidentified taxon	Castle Garden	Fully quantified	12.753	14.938	117.13	B01
FB005	Unknown/unidentified taxon	Castle Garden	Fully quantified	11.377	19.000	167.00	B01
FB006	Unknown/unidentified taxon	Castle Garden	Fully quantified	3.910	1.986	50.80	B01
FB007	Unknown/unidentified taxon	Castle Garden	Fully quantified	9.332	14.605	156.49	B01
FB008	Unknown/unidentified taxon	Castle Garden	Fully quantified	5.208	4.269	81.96	B01
FB010	Unknown/unidentified taxon	Castle Garden	Fully quantified	21.496	31.165	144.98	B01
FB011	Unknown/unidentified taxon	Castle Garden	Fully quantified	7.598	5.916	77.87	B02
FB014	Unknown/unidentified taxon	Castle Garden	Fully quantified	4.366	5.032	115.24	B02
FB015	Unknown/unidentified taxon	Castle Garden	Fully quantified	10.357	11.102	107.19	B02
FB016	Unknown/unidentified taxon	Castle Garden	Fully quantified	6.758	7.746	114.62	B02
FB017	Unknown/unidentified taxon	Castle Garden	Fully quantified	16.156	8.013	49.60	B02
FB019	*Panaeolina foenisecii*	Abbey Park	Fully quantified	163.705	226.832	138.56	B05
FB086	*Mycena citrinomarginata*	Victoria Park	Fully quantified	45.856	54.896	119.71	B10
FB087	*Mycena citrinomarginata*	Victoria Park	Fully quantified	65.013	66.847	102.82	B10
FB088	*Mycena citrinomarginata*	Victoria Park	Fully quantified	22.414	22.869	102.03	B10
FB090	*Agaricus arvensis*	Fosse Rec. Ground	Partially quantified	2.646–2.933	—	—	B05
FB092	*Agaricus bitorquis*	St Augustine Road	Partially quantified	1.763–2.859	—	—	B02
FB093	*Agaricus bitorquis*	St Augustine Road	Fully quantified	10.681	7.934 ^1^	cap 113.46; stem 74.79	B02
FB094	*Agaricus bitorquis*	St Augustine Road	Partially quantified	2.614–3.705	—	—	B03
FB095	*Agaricus bitorquis*	St Augustine Road	Partially quantified	1.947–3.041	—	—	B03
FB101	*Agaricus bitorquis*	St Augustine Road	Partially quantified	6.900–7.987	—	—	B03
FB102	*Agaricus bitorquis*	St Augustine Road	Fully quantified	10.443	5.438 ^1^	cap 183.07; stem 44.22	B03; B04
FB105	*Agaricus bitorquis*	St Augustine Road	Partially quantified	1.385–1.673	—	—	B04
FB111	*Agaricus bitorquis*	St Augustine Road	Partially quantified	1.698–1.987	—	—	B05

Fully quantified range: 3.91–163.70 ng g^−1^ dw. The interval-censored lognormal model estimated a population median of 1.055 ng g^−1^ dw (95% bootstrap CI: 0.672–1.571; all 1000 bootstrap fits converged successfully; 73 left-censored, 28 interval-censored and 20 exact observations). Any signal above the operational detection threshold: 48/121 (39.7%); at least one quantified component: 27/121 (22.3%). For fully quantified units represented by one analytical record, the archived Syngistix ^187^Re RSD represents variability across five instrumental replicate measurements, and the corresponding instrumental SD was derived from the unrounded dry-weight Re concentration × RSD/100. Because conversion from introduced-solution concentration to dry-weight concentration is linear, relative precision is preserved during this transformation. ^1^ For the two fully quantified cap–stem units (FB093 and FB102), component-specific instrumental SDs were propagated through the same dry-mass-weighted reconstruction used for the concentration estimate, assuming independent instrumental variation between the separately analysed components; cap and stem RSDs are shown separately. Partially quantified units remain interval-censored and were not assigned an SD. SD and RSD describe within-run instrumental precision and not total procedural or method uncertainty. The five individual replicate concentrations were not retained in the archived dataset.

**Table 3:** Published Re concentrations in mushroom fruiting bodies relevant to interpretation of the present dataset.

Study	Mushroom Material/Context	Sampling/*N*	Re Result	Comparability Note
Present study	Wild mushrooms; Leicester/Leicestershire, UK	121 reconstructed fruiting-body units	0.00391–0.16370 mg kg^−1^ dw in fully quantified units; model-based population median 0.001055 mg kg^−1^ dw	ICP–MS; censoring-aware analysis; public green-space monitoring
Mleczek et al. (2016) [11]	20 wild species near a heavily trafficked road, Poland	10 soil-growing + 10 wood-growing species; fruiting bodies analysed in triplicate	*Tricholoma equestre*: 0.21 ± 0.05 mg kg^−1^ dw, the highest reported aboveground value	ICP–OES; roadside setting; comparative Re literature noted as sparse
Mleczek et al. (2017) [12]	Commercial *Lentinula edodes*, Poland	Commercially cultivated samples, 2007–2015	0.24 ± 0.11 mg kg^−1^ dw (general mean)	Cultivated food mushroom; multielement analysis
Safonov et al. (2020) [13]	Zhireken Cu–Mo mineralised area	Individual reported fungal samples	*Leccinum versipelle* cap 0.23; *Xerocomus subtomentosus* 0.054; *Suillus elegans* 1.91 mg kg^−1^ dw	Ore-associated/mineralised setting; geochemical comparator, not environmental background
Busuioc et al. (2008) [14]	*Macrolepiota procera* and *Armillaria/Armillariella mellea*	Exploratory fungal survey	Re occurrence reported; no directly comparable quantitative benchmark retained here	Historical/contextual evidence; analytical and reporting comparability limited

Values from mineralised areas are retained as geochemical comparators and should not be interpreted as environmental background. Busuioc et al. [14] is retained as contextual/historical evidence rather than as a directly comparable quantitative benchmark.

## Data Availability

The data presented in this study are available in the Open Science Framework (OSF) repository, *Leicester topsoil and wild mushroom metal dataset*, at https://osf.io/xbrd7/overview?view_only=750aba9f380044e38ce5c9162e0d4020 (accessed on 15 August 2026). The repository includes curated CSV files containing raw and final calculated concentrations, limits of detection (LoDs) and below-LoD flags for the topsoil and wild mushroom datasets. The dataset is released under the Creative Commons Attribution 4.0 International licence (CC BY 4.0).

## Supplementary Materials

The following supporting information can be downloaded at https://www.mdpi.com/article/10.3390/ijms27188405/s1.
