# Supplementary material for: Rhenium in Wild Mushrooms: Environmental Occurrence and Censoring-Aware Quantification
Source: Int J Mol Sci. 2026 Sep 21;27(18):8405. doi: 10.3390/ijms27188405 (PMC13607020; doi:10.3390/ijms27188405)
Supplement: Supplementary file 1 [file ijms-27-08405-s001.zip › ijms-4541130-supplementary.pdf]

Table S1. Detailed Re calibration performance and selection of the study-specific operational LLOQ.

Level	Nominal (μg L^{-1})	Apparent (μg L^{-1})	RSD (%)	Back-calculation error (%)	Role
Cal1	0.005	0.005	7.15	−14.05	Selected operational LLOQ
Cal2	0.011	0.010	3.56	−6.04	
Cal3	0.027	0.027	4.81	−0.41	
Cal4	0.055	0.057	3.09	+4.37	
Cal5	0.137	0.137	2.30	−0.62	Upper calibration level

Calibration R^2 = 0.999476. The lowest calibrator was retained as the study-specific operational LLOQ.

Table S2. Quantitatively informative Re fruiting-body units under Scenario A, with archived instrumental-precision information for fully quantified analytical records.

Unit	Final taxon	Park	Classification	Re result (ng g^{-1} dw)	Analytical record(s)	Archived ^{187}Re RSD (%)	Archived ^{115}In RSD (%)	Batch
FB001	Unknown/unidentified taxon	Castle Garden	Fully quantified	9.786	Mushroom 016	128.77	0.86	B01
FB002	Unknown/unidentified taxon	Castle Garden	Fully quantified	12.657	Mushroom 017	107.12	2.29	B01
FB003	Unknown/unidentified taxon	Castle Garden	Fully quantified	4.122	Mushroom 018	84.62	2.33	B01
FB004	Unknown/unidentified taxon	Castle Garden	Fully quantified	12.753	Mushroom 019	117.13	1.73	B01
FB005	Unknown/unidentified taxon	Castle Garden	Fully quantified	11.377	Mushroom 020	167.00	1.61	B01
FB006	Unknown/unidentified taxon	Castle Garden	Fully quantified	3.910	Mushroom 021	50.80	4.08	B01
FB007	Unknown/unidentified taxon	Castle Garden	Fully quantified	9.332	Mushroom 022	156.49	3.05	B01
FB008	Unknown/unidentified taxon	Castle Garden	Fully quantified	5.208	Mushroom 023	81.96	4.17	B01
FB010	Unknown/unidentified taxon	Castle Garden	Fully quantified	21.496	Mushroom 025	144.98	1.60	B01
FB011	Unknown/unidentified taxon	Castle Garden	Fully quantified	7.598	Mushroom 027	77.87	2.00	B02
FB014	Unknown/unidentified taxon	Castle Garden	Fully quantified	4.366	Mushroom 032	115.24	1.48	B02
FB015	Unknown/unidentified taxon	Castle Garden	Fully quantified	10.357	Mushroom 033	107.19	0.90	B02
FB016	Unknown/unidentified taxon	Castle Garden	Fully quantified	6.758	Mushroom 034	114.62	2.73	B02
FB017	Unknown/unidentified taxon	Castle Garden	Fully quantified	16.156	Mushroom 035	49.60	3.42	B02
FB019	*Panaeolina foenisecii*	Abbey Park	Fully quantified	163.705	Mushroom 094	138.56	1.30	B05
FB086	*Mycena citrinomarginata*	Victoria Park	Fully quantified	45.856	Mushroom 192	119.71	1.44	B10
FB087	*Mycena citrinomarginata*	Victoria Park	Fully quantified	65.013	Mushroom 193	102.82	0.55	B10
FB088	*Mycena citrinomarginata*	Victoria Park	Fully quantified	22.414	Mushroom 194	102.03	0.92	B10
FB090	*Agaricus arvensis*	Fosse Rec. Ground	Partially quantified	2.646–2.933	—	—	—	B05
FB092	*Agaricus bitorquis*	St Augustine Road	Partially quantified	1.763–2.859	—	—	—	B02
FB093	*Agaricus bitorquis*	St Augustine Road	Fully quantified	10.681	Cap: Mushroom 039; Stem: Mushroom 040	cap 113.46; stem 74.79	cap 2.29; stem 1.88	B02
FB094	*Agaricus bitorquis*	St Augustine Road	Partially quantified	2.614–3.705	—	—	—	B03

Unit	Final taxon	Park	Classification	Re result (ng g^{-1} dw)	Analytical record(s)	Archived ^{187}Re RSD (%)	Archived ^{115}In RSD (%)	Batch
FB095	*Agaricus bitorquis*	St Augustine Road	Partially quantified	1.947–3.041	—	—	—	B03
FB101	*Agaricus bitorquis*	St Augustine Road	Partially quantified	6.900–7.987	—	—	—	B03
FB102	*Agaricus bitorquis*	St Augustine Road	Fully quantified	10.443	Cap: Mushroom 060; Stem: Mushroom 057	cap 183.07; stem 44.22	cap 2.59; stem 1.42	B03;B04
FB105	*Agaricus bitorquis*	St Augustine Road	Partially quantified	1.385–1.673	—	—	—	B04
FB111	*Agaricus bitorquis*	St Augustine Road	Partially quantified	1.698–1.987	—	—	—	B05

Fully quantified units are exact fruiting-body-level values; partially quantified units are represented by lower–upper analytical intervals and are not reduced to midpoint substitutions. The archived ^{187}Re and ^{115}In RSD values are Syngistix within-run instrumental precision statistics from five instrumental replicate measurements. Across the 22 analytical component records contributing to the 20 fully quantified units, ^{187}Re RSDs ranged from 44.22% to 183.07% (median 110.32%; mean 108.28%), whereas ^{115}In RSDs ranged from 0.55% to 4.17% (median 1.81%; mean 2.03%). Sample-specific SD values and the five individual replicate concentrations were not separately retained in the archived workbook. For FB093 and FB102, component-specific cap and stem RSDs are shown separately; no unit-level RSD or SD was reconstructed. These RSD values describe within-run instrumental repeatability and not total procedural or method uncertainty.

Table S3. Descriptive Re occurrence profiles by collection location.

Location	*N*	ND	Detected below operational LLOQ	Partially quantified	Fully quantified	Any signal (%)	Any quantified (%)	Batches
Abbey Park	23	19	3	0	1	17.4	4.3	B05;B06;B07
Beaumont Leys Park	1	1	0	0	0	0.0	0.0	B07
Bradgate Park	8	8	0	0	0	0.0	0.0	B09
Braunstone Park	5	5	0	0	0	0.0	0.0	B07;B08
Castle Garden	17	0	3	0	14	100.0	82.4	B01;B02
Fosse Rec. Ground	1	0	0	1	0	100.0	100.0	B05
Jesse Jackson Park	7	7	0	0	0	0.0	0.0	B08
Knighton Park	4	4	0	0	0	0.0	0.0	B08
Portland Building, DMU	1	1	0	0	0	0.0	0.0	B10
St Augustine Road	22	1	13	6	2	95.5	36.4	B02;B03;B04;B05
Stokes Wood Park	7	7	0	0	0	0.0	0.0	B07
Victoria Park	22	19	0	0	3	13.6	13.6	B09;B10
Western Park	3	1	2	0	0	66.7	0.0	B05

Descriptive only. Sampling intensity, taxonomic composition and analytical-batch representation were unbalanced across locations.

Table S4. Descriptive Re occurrence profiles by final taxonomic assignment.

Final taxon	*N*	ND	Detected below operational LLOQ	Partially quantified	Fully quantified	Any signal (%)	Any quantified (%)	Batches
Agaricus arvensis	2	0	1	1	0	100.0	50.0	B05
Agaricus bitorquis	22	1	13	6	2	95.5	36.4	B02;B03;B04;B05
Agaricus nivescens	1	1	0	0	0	0.0	0.0	B08
Armillaria mellea	3	3	0	0	0	0.0	0.0	B08
Coprinopsis atramentaria	8	8	0	0	0	0.0	0.0	B09
Hypholoma fasciculare	1	1	0	0	0	0.0	0.0	B08
Leucoagaricus leucothites	1	1	0	0	0	0.0	0.0	B10
Marasmius oreades	4	4	0	0	0	0.0	0.0	B08
Mycena citrinomarginata	22	19	0	0	3	13.6	13.6	B09;B10
Panaeolina foenisecii	28	24	3	0	1	14.3	3.6	B05;B06;B07;B08
Pholiota adiposa	2	2	0	0	0	0.0	0.0	B07
Pholiota gummosa	1	1	0	0	0	0.0	0.0	B07
Psathyrella corrugis	1	1	0	0	0	0.0	0.0	B07
Psathyrella fatua	3	3	0	0	0	0.0	0.0	B07
Psathyrella sublatispora	1	1	0	0	0	0.0	0.0	B07
Vascellum pratense	2	2	0	0	0	0.0	0.0	B08
Xerocomus porosporus	2	1	1	0	0	50.0	0.0	B05

Taxon profiles are descriptive because several taxa are concentrated within individual locations and/or analytical batches. The 17 unknown/unidentified units are excluded from named-taxon biological inference.

Table S5. Eligibility of matched cap–stem fruiting bodies for tissue-specific quantitative comparison.

Cap-stem reconstruction metric	*n*	Interpretation
Matched cap-stem fruiting-body units	32	Two analytical portions per reconstructed fruiting body
Both cap and stem quantitatively exact	2	Only these pairs support an exact cap-stem concentration comparison
One component quantitatively exact	7	Fruiting-body reconstruction remains interval/partially quantified
No quantitatively exact component	23	No exact cap-stem concentration comparison

Because fewer than three matched pairs had exact concentrations in both tissues, no inferential cap-versus-stem comparison was performed.

Table S6. Sensitivity of Re analytical classification to alternative background scenarios.

Scenario	Analytical basis	Evaluable *N*	ND	Detected below operational LLOQ	Partially quantified	Fully quantified	Not evaluable
A	Primary: post-replacement procedural-blank population	121	73	21	7	20	0
B	Strict post-replacement same-batch sensitivity	59	56	0	0	3	62
C	All 18 archived acid-digestion blanks	121	120	0	1	0	0
D	Wash-only instrumental diagnostic	121	73	22	6	20	0

Scenario A–B agreement was 59/59 (100%) among directly comparable units; 62 units lacked an eligible post-replacement same-batch procedural blank. Scenario C intentionally includes the earlier procedural phase and is a conservative sensitivity boundary.

Table S7. Contextual composite-topsoil dataset, Re analytical status and site-level mushroom–soil matching.

**Item**	**Status / interpretation**
Environmental unit	26 homogenised site-level composite topsoils.
Field sampling basis	850 surface-soil increments pooled within sampling locations and homogenised to generate the 26 composites.
QA/QC representation	52 physical subsamples (two per composite), retained as laboratory/preparation replication while preserving N = 26 environmental units.
Soil CRM	CRM059-50G Trace Metals–Loamy Clay 2.
Broader soil CRM recovery	94.9–108.6% for elements with assigned values [41].
Re in CRM059	No assigned Re mass fraction; Re-specific soil recovery not calculable.
Operational Re detection threshold	0.0718 μg L^{-1} on the 11-fold-diluted analytical-solution scale, corresponding to approximately 79.0 ng g^{-1} dw under the documented soil-preparation conditions.
Re status in topsoil dataset	All 52 physical topsoil subsamples were below the operational Re detection threshold; therefore, no quantitatively resolved Re concentration could be assigned to any of the 26 site-level composites.
Eligible mushroom–soil site matches	Nine mushroom-collection sites had corresponding site-level composites based on exact site-name matching or previously validated aliases: Abbey Park, Bradgate Park, Braunstone Park, Castle Garden(s), Knighton Park, Stokes Wood Park, Victoria Park, Western Park and Beaumont Leys/Beaumont Park.
Matched soil result	All nine matched site-level topsoil composites were below the operational Re detection threshold.
Mushroom Re at matched sites	Mushroom-associated Re signals occurred at four of the nine matched sites: Castle Garden, Abbey Park, Victoria Park and Western Park.
Interpretive use	Park-scale geochemical context and exploratory site-level matching only. The composites were not collected directly beneath individual fruiting bodies and do not constitute microscale soil–fungus pairs. Because the soil Re dataset was entirely left-censored at the operational threshold, quantitative soil–mushroom correlations, concentration ratios, bioconcentration factors and transfer factors were not calculated.

The soil non-detections should not be interpreted as evidence that Re was absent from the park soils; the soil-method threshold was substantially higher than the concentration range relevant to much of the fungal dataset.

Table S8. Detailed molecular and toxicological literature context for chemically defined Re species and its relevance to interpretation of environmental total-Re occurrence.

**Re form / compound**	**Experimental model**	**Exposure / uptake feature**	**Molecular / cellular endpoint**	**Main mechanistic finding**	**Relevance to environmental Re**
Perrhenate (ReO_4^-) [17]	In vivo NIS-expressing tissues	NIS-mediated uptake and biodistribution	Tissue uptake and clearance	Perrhenate is transported by the sodium–iodide symporter; biodistribution demonstrates biological handling of a soluble Re(VII) oxyanion	High conceptual relevance to biological transport; not an environmental toxicity benchmark
Re(I) tricarbonyl complexes [22]	Cancer cell models	Mitochondrial accumulation	ROS generation and glutathione disruption	Irreversible oxidative stress and cell-death pathways were induced at pharmacological experimental exposures	Mechanistic evidence only; chemical form and dose differ fundamentally from mushroom Re
Mitochondria-immobilised Re(I) complex [23]	Cancer cell models	Mitochondrial localisation	Respiration, ATP, ROS and apoptosis	Suppressed mitochondrial metabolism with reduced ATP and increased oxidative stress	Mechanistic evidence only
DFX–Re(I) complex [24]	Cellular and tumour models	Mitochondrial targeting / iron-homeostasis perturbation	DNA, RNA and histone methylation	Metabolic and iron-homeostasis intervention produced epigenetic reprogramming	Mechanistic evidence only; not transferable to stable elemental Re occurrence
Re isonitrile complex [25]	Cancer cell models	Cellular internalisation	UPR/eIF2α–ATF4–CHOP signalling; mitochondrial fission	Endoplasmic-reticulum stress and unfolded-protein-response-mediated apoptosis	Mechanistic evidence only
Artesunate–Re(I) complexes [26]	Cancer cell models	Mitochondrial targeting	GSH/GPX4, lipid peroxidation, apoptosis and ferroptosis	Dual apoptosis–ferroptosis mechanisms depended on designed molecular structure	Mechanistic evidence only
Octahedral / triazole Re_6 clusters [27,28]	Cell models	Structure-dependent internalisation	DNA binding and cytotoxicity	Cellular uptake and toxicity varied substantially with ligand/cluster structure	Strong evidence that biological behaviour is species/structure dependent

Re form / compound	Experimental model	Exposure / uptake feature	Molecular / cellular endpoint	Main mechanistic finding	Relevance to environmental Re
Tricarbonyl Re complexes [29]	Cancer cell models	Intracellular pharmacological exposure	G2/M arrest; Aurora-A kinase phosphorylation	Antiproliferative activity involved cell-cycle and kinase signalling	Mechanistic evidence only

The molecular evidence concerns chemically defined Re species and pharmacological experimental systems. It provides mechanistic context for future investigation of environmentally encountered Re species and should not be extrapolated quantitatively to the total Re concentrations measured in the present mushroom samples.
